# Exploring Dacarbazine Complexation with a Cellobiose-Based Carrier: A Multimethod Theoretical, NMR, and Thermochemical Study

**DOI:** 10.3390/molecules30244819

**Published:** 2025-12-18

**Authors:** Marta Hoelm, Zdzisław Kinart, Stanisław Porwański

**Affiliations:** 1 University of Lodz, Faculty of Chemistry, Department of Physical Chemistry, Pomorska 163/165, 90-236 Lodz, Poland; zdzislaw.kinart@chemia.uni.lodz.pl; 2 University of Lodz, Faculty of Chemistry, Department of Organic and Applied Chemistry, Tamka 12, 91-403 Lodz, Poland; stanislaw.porwanski@chemia.uni.lodz.pl

**Keywords:** sugar cryptand, drug delivery, enthalpy, Gibbs free energy, DFT calculations, NMR spectroscopy, hydrogen bonds

## Abstract

Dacarbazine (DTIC) is a clinically important anticancer drug whose photosensitivity poses challenges for its stability and interactions with supramolecular hosts. Here, we investigate its complexation with the host 1,10-*N,N′*-bis-(β-D-ureidocellobiosyl)-4,7,13,16-tetraoxa-1,10-diazacyclooctadecane (TN), a hybrid urea–carbohydrate–diazacrown system, using combined experimental and computational approaches. While TN has been studied as a host molecule, its specific interactions with DTIC and the associated thermodynamic characteristics had not been characterized. Computational results (obtained at the density functional theory level (DFT)) indicate that TN primarily forms non-inclusion complexes, with DTIC engaging in hydrogen bonding with sugar units, urea bridges, and diazacrown ether moieties. Experimental ^1^H NMR studies in D_2_O confirmed these interaction patterns, showing notable chemical shifts for sugar protons. Conductometric measurements between 293 and 313 K allowed for the determination of formation constants and thermodynamic parameters. The results demonstrate that TN:DTIC complexation is spontaneous, exothermic, and enthalpy-driven, accompanied by decreased system entropy. Comparison with previous studies on cyclodextrin complexes shows that TN forms strong associations with DTIC, owing to its abundant donor–acceptor groups, which facilitate extensive hydrogen-bonding networks. These findings provide new insights into DTIC stabilization and highlight TN’s potential as a multifunctional platform for drug delivery.

## 1. Introduction

Dacarbazine (DTIC) is a purine-like imidazole carboxamide derivative that belongs to the class of triazene alkylating agents [1]. It exerts its anticancer activity primarily via DNA alkylation, leading to genetic damage and subsequent cancer cell death [2]. Dacarbazine was approved by the U.S. Food and Drug Administration (FDA) as the first and only chemotherapeutic agent for the treatment of malignant melanoma [3]. Historically, dacarbazine, together with doxorubicin and ifosfamide, constituted one of the principal systemic treatments for patients with unresectable or metastatic soft tissue sarcoma (STS) [4].

The cytotoxic mechanism of dacarbazine has been linked not only to its alkylating activity but also to the generation of reactive oxygen species (ROS), including hydrogen peroxide (H_2_O_2_) and superoxide anion (O_2_^−^). These reactive intermediates induce lysosomal membrane permeabilization, the release of proteolytic enzymes, and ultimately apoptotic or necrotic cell death [5].

Despite its potent antitumor effects, dacarbazine demonstrates unsatisfactory clinical efficacy. Reported response rates vary between 10% and 25%, and complete remission is observed in fewer than 5% of patients [6]. The therapeutic potential of DTIC is further limited by its poor aqueous solubility, short plasma half-life (approximately 41 min), and systemic toxicity manifested by vomiting, neutropenia, myelosuppression, and alopecia [7]. DTIC may also induce hepatotoxicity, especially when combined with other anticancer agents [8]. In addition, tumor resistance to dacarbazine is frequently associated with the upregulation of the DNA repair enzyme O^6^-methylguanine-DNA methyltransferase (MGMT), which counteracts DNA alkylation and reduces treatment efficacy [9].

Clinically, dacarbazine is administered either as a monotherapy or in various multidrug regimens such as doxorubicin–dacarbazine or dacarbazine–fluorouracil [10,11]. The recommended intravenous dose ranges between 0.9 and 1.0 g/m^2^ every three weeks [12]. However, newer therapeutic agents such as eribulin have demonstrated improved survival outcomes in advanced soft tissue sarcomas, suggesting the need for more effective alternatives [13].

To address the pharmacokinetic and physicochemical drawbacks of DTIC, numerous formulation strategies have been explored. These include the development of novel salt and cocrystal forms that improve solubility and dissolution behavior [14], as well as a variety of nanotechnology-based delivery systems designed for targeted and sustained release. Nanoparticles, liposomes, solid lipid carriers, nanosponges, and polymeric nanogels have all been investigated for dacarbazine encapsulation, demonstrating enhanced bioavailability, tumor selectivity, and reduced systemic toxicity [15,16,17]. Furthermore, co-delivery systems combining dacarbazine with bioactive molecules such as miRNA-34a or ursolic acid have produced synergistic cytotoxic effects compared with dacarbazine monotherapy [18].

Drug delivery systems involving DTIC have also been analyzed using theoretical approaches. For example, in reference [19], the beryllium oxide (Be_12_O_12_) nanocarrier was examined as a potential and effective carrier. The authors employed density functional theory (DFT) to calculate adsorption and interaction energies. The computed results showed negative adsorption and interaction energy values for all considered configurations, indicating that the formation of such systems is energetically favorable. In another study [20], the influence of Al, Ga, and In dopants on the dacarbazine delivery performance of a BC_3_ nanosheet (BCNS) was investigated using various theoretical methods. It was found that pristine BCNS is not suitable for this type of drug delivery, as incorporating Al, Ga, or In atoms into the BCNS surface significantly increases the adsorption energy of dacarbazine.

Given these ongoing challenges, the search for novel, biocompatible, and effective drug delivery systems (DDS) remains an active field of research. Beyond conventional carriers such as cyclodextrins [21], liposomes [22], or polymeric nanoparticles [23], sugar-based macrocyclic cryptands have emerged as promising alternatives [24,25,26]. These compounds can form stable supramolecular complexes with drug molecules, improving their solubility and stability. In this context, the diazacrown cryptand 1,10-*N,N′*-bis-(β-D-ureidocellobiosyl)-4,7,13,16-tetraoxa-1,10-diazacyclooctadecane (TN) has recently been studied as a carrier for alkylating agents, such as carmustine [25] and mitomycin C [26].

**Figure 1:**
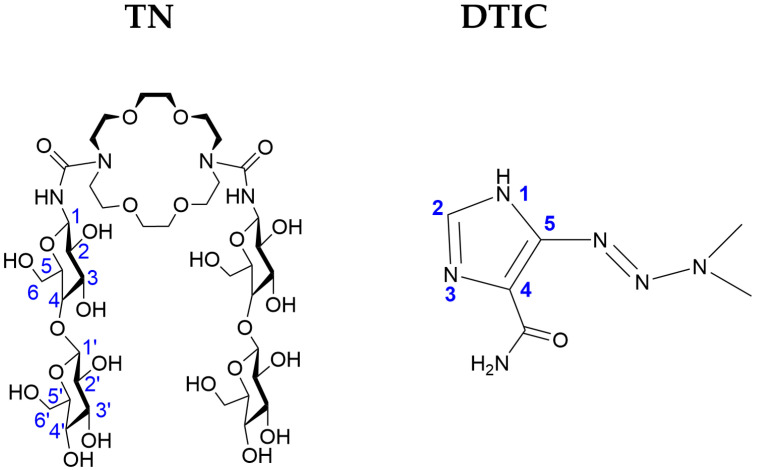
Schematic representation of 1,10-*N,N′*-bis-(β-D-ureidocellobiosyl)-4,7,13,16-tetraoxa-1,10-diazacyclooctadecane (TN) and dacarbazine (DTIC) with numbering used in the NMR analysis.

In our previous work [27], we investigated the complexation stability of various cyclodextrins with DTIC and found that hydroxyethyl-β-cyclodextrin forms the most stable complex. In the present study, we examined and compared the complexation abilities of TN (Figure 1) toward DTIC (Figure 1) in aqueous solution. The analysis was carried out using theoretical, spectroscopic, and thermodynamic approaches. Our results demonstrate that TN is capable of forming a stable, non-inclusion complex with DTIC, in which the drug is bound to TN through hydrogen bonds. Conductometric measurements further indicate that the complex is thermodynamically stable. Compared to previous studies on cyclodextrin complexes, TN exhibits strong binding with DTIC, owing to its numerous donor–acceptor groups that facilitate extensive hydrogen-bonding networks.

## 2. Results and Discussion

### 2.1. Analysis of Structural and Energetic Properties of the TN:DTIC Complex-Theoretical Study

**Figure 2:**
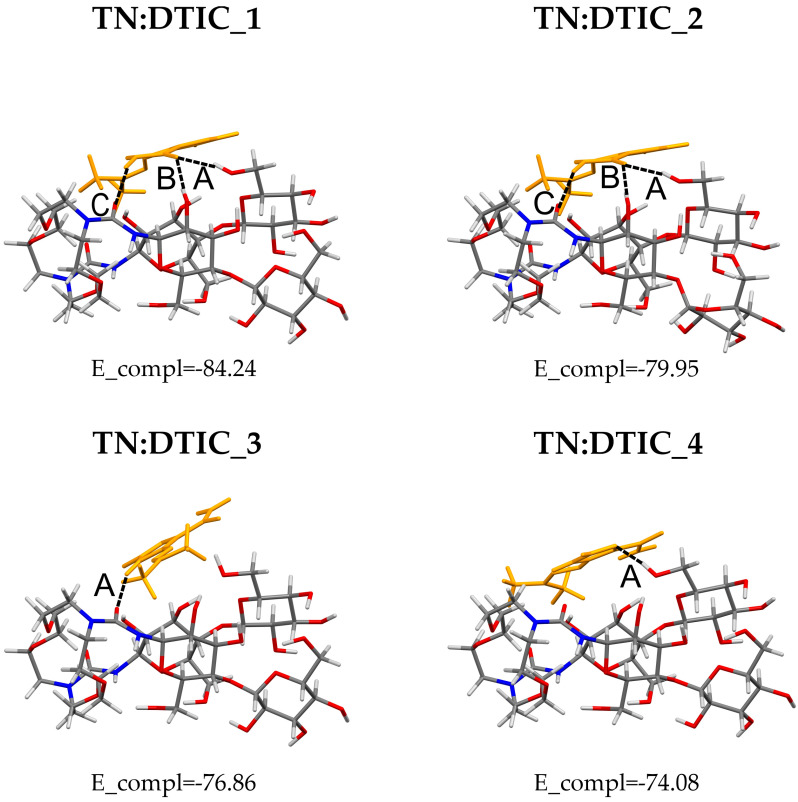
The most stable complexes, obtained from M08-HX-D3/6-31G(d,p) calculations in water (PCM), with their complexation energies (E_compl) reported in kJ/mol. Dotted lines labeled with letters indicate hydrogen bonds. Atom colors: carbon—grey, nitrogen—dark blue, oxygen—red, hydrogen—light grey; DTIC—orange.

The most stable complexes identified in the final step of the configurational search at the density functional theory (DFT) level are shown in Figure 2. Their total energy values are listed in Table S1 in the Electronic Supplementary Information (ESI), and the corresponding coordinates are provided in Table S2. As illustrated in Figure 2, only non-inclusion complexes are displayed, in which DTIC is bound to the external surface of TN. Although inclusion models of the complex were initially constructed, they were disrupted during optimization, with some atoms detaching. It should be noted that a similar behavior was observed in our previous work [25,26], where we investigated the possibility of forming inclusion complexes between TN and carmustine or mitomycin C. In those cases, TN was also unable to adopt such geometries. This limitation likely arises from the specific geometry of isolated TN, which forms a compact structure stabilized by numerous hydrogen bonds (HB) between its sugar units. In the most stable conformer of TN (TN-1), eight intramolecular hydrogen bonds were identified [25]. Formation of an inclusion complex would disrupt this hydrogen-bonding network, thereby increasing the total energy of the structure. Moreover, TN does not possess a well-defined internal cavity, such as those present in cyclodextrins [28] or nanotubes [29].

As shown in Figure 2, DTIC interacts with all fragments of TN, including the sugar units, diazacrown ether, and urea bridges. In these complexes, both moderate and weak hydrogen bonds were observed, classified according to Jeffrey’s criteria based on the H···A distance, where H represents the hydrogen atom and A the acceptor [30]. To avoid overcrowding Figure, only hydrogen bonds of moderate strength are shown in Figure 2. The geometrical parameters of HB are listed in Table S3.

An interesting situation is observed in the TN:DTIC_1 and TN:DTIC_2 complexes, which appear similar in geometry (RMSD = 0.4 Å). However, one of the more distant glucose residues (with respect to the crown ether) in TN:DTIC_2 is more deformed than in TN:DTIC_1. This deformation significantly affects the complexation energy—the difference in E_compl between TN:DTIC_1 and TN:DTIC_2 amounts to 4.29 kJ/mol- even though this glucose unit does not directly participate in, for instance, hydrogen bonding with DTIC.

**Figure 3:**
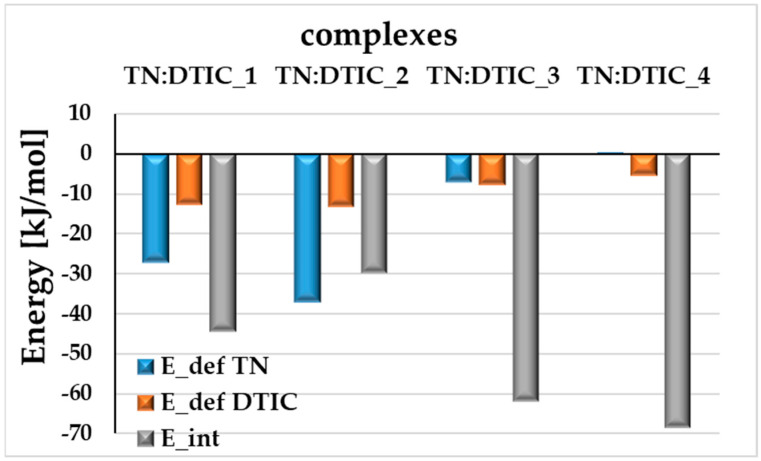
Energetic parameters: deformation energies of TN (E_def TN) and DTIC (E_def DTIC), and interaction energies (E_int) obtained at the M08-HX-D3/6-31G(d,p) level of theory in water (PCM model).

It is worth considering why these complexes (presented in Figure 2) are the most stable. Maybe a key factor is that their geometries allow for the formation of a substantial number of hydrogen bonds. In each complex, TN retains 6–7 moderate intramolecular hydrogen bonds, nearly the same as in isolated TN, which has eight [25]. The number of intermolecular hydrogen bonds may also correlate with stability: the two most stable complexes, TN:DTIC_1 and TN:DTIC_2, each form three intermolecular hydrogen bonds, whereas the less stable complexes form only one. The formation of these hydrogen bonds may also have a direct impact on the deformation energies. As shown in Figure 3, the deformation energy of TN (E_def TN) is significantly higher than that of DTIC (E_def DTIC) in TN:DTIC_1 and TN:DTIC_2. The stronger deformations in these two complexes reflect the energetic cost of optimizing TN’s geometry to maximize hydrogen-bonding interactions with DTIC. In the less stable complexes, where only one hydrogen bond is formed, the deformation energies are smaller and the trend is reversed. Interestingly, in TN:DTIC_4, E_def TN is slightly positive (~0.0038 kJ/mol), indicating that TN’s geometry becomes slightly more energetically favorable upon complexation compared to its isolated form. The most stable complexes, which exhibit the lowest complexation energies and the highest deformation energies, contribute to weaker overall interactions. In the case of TN:DTIC_1 and TN:DTIC_2, the substantial deformation of TN reduces the effective interaction strength between the components of the complex.

It is worth comparing the complexation ability of TN toward DTIC with literature data. TN has been investigated by us previously as a potential drug carrier for carmustine (BCNU) [25] and mitomycin C (MMC) [26]. In both studies, we analyzed the structural, energetic, spectroscopic, and biological properties of the resulting complexes. Some aspects of TN’s complexation behavior remain consistent regardless of the drug analyzed. TN primarily forms non-inclusion complexes, in which the drug is bound to the external surface of TN. The interactions are mainly hydrogen bonds formed with the sugar units of TN. The complexation stability of TN:BCNU was found to be approximately −85 kJ/mol, while that of TN:MMC was about −108 kJ/mol. However, it should be noted that both values were obtained at a different level of theory than the one used in this work, namely M06-2X-D3/6-31G(d,p).

A direct comparison can only be made between complexation energies calculated at the same theoretical level. Such an analysis was performed in our previous study [27], where various cyclodextrins were investigated as potential carriers for dacarbazine. In that work, the reported complexation energies were not corrected for BSSE. Accordingly, the complexation energy for TN:DTIC_1 without BSSE correction equals −104 kJ/mol, which corresponds, in terms of stability, to the complexation energy obtained for HP-β-CD (~−106 kJ/mol). However, it should be emphasized that the latter value corresponds to an inclusion complex. Therefore, it is difficult to estimate what the E_compl value would be for a non-inclusion HP-β-CD:DTIC complex. Although such geometries are less common, as reported in reference [31], this type of complex is significantly less stable than the inclusion one.

### 2.2. Analysis of Spectroscopic Properties of Complex-Comparison Between Theoretical and Experimental Results

**Figure 4:**
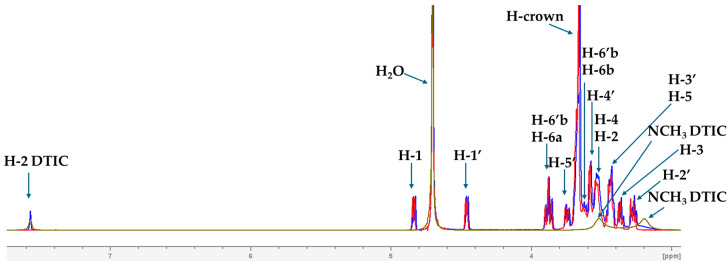
Overlapped ^1^H NMR spectra recorded in D_2_O solution. The spectrum of the complex is shown in blue, that of dacarbazine in green, and TN in red.

**Figure 5:**
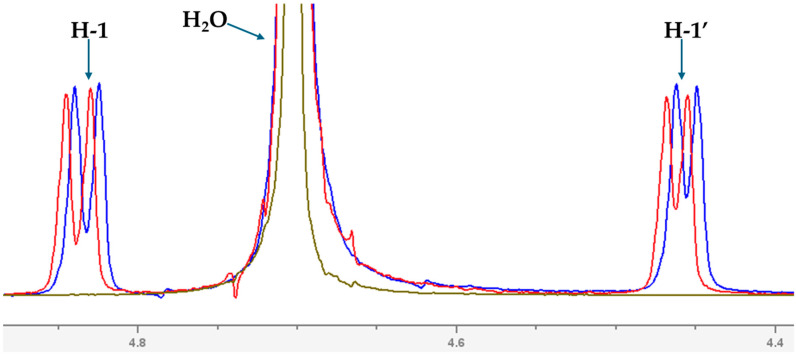
Fragment of the ^1^H NMR spectrum showing the H-1 and H-1′ signals corresponding to the cellobiose units of TN.

**Figure 6:**
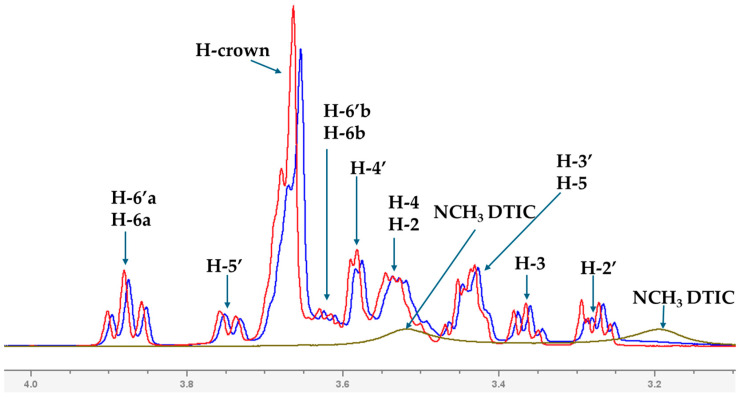
Fragment of the ^1^H NMR spectrum showing the signals corresponding to the diazacrown ether and sugar units of TN, as well as the protons belonging to dacarbazine (NCH_3_).

The formation of the complex was investigated using ^1^H Nuclear Magnetic Resonance (NMR) spectroscopy. The experimental NMR measurements were carried out in heavy water (D_2_O), whereas the theoretical calculations were performed in water using a larger basis set (M08-HX/6-31++G(d,p)). Figure 4 presents the overlapping NMR spectra, allowing visualization of the chemical shift changes (Δδ) upon complex formation. ^1^H NMR spectra for the isolated DTIC and TN are shown in Figure S1 and Figure S2, respectively. As can be seen, the Δδ values are generally small; however, more pronounced shifts are observed for the H-1 and H-1′ protons (Figure 5), as well as for protons belonging to the sugar units and crown ether (Figure 6). In turn, in the case of DTIC, significant changes are observed for the protons of the −NCH_3_ groups. The chemical shift values and their changes upon complex formation are summarized in Table S4. The COSY spectra of isolated DTIC and TN are shown in Figure S3 and Figure S4, respectively, and the corresponding spectrum of the complex is provided in Figure S5. According to the computational results, DTIC primarily forms hydrogen bonds with the hydroxyl groups of the cellobiose units in TN. Unfortunately, due to the solvent used in the NMR experiments, the chemical shift in the hydroxyl protons are not observable.

**Figure 7:**
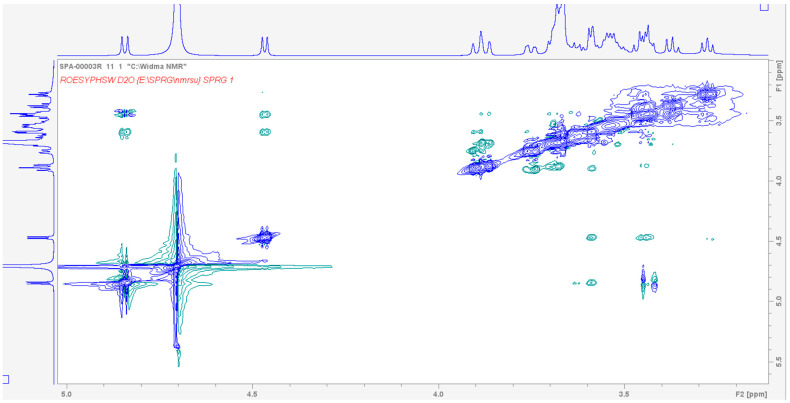
The fragment of the ROESY spectrum of the TN:DTIC complex showing the region of cellobiose units of TN and protons of DTIC.

In Figure 7, the ROESY (Rotating-frame Overhauser Effect Spectroscopy) spectrum is shown. This technique reveals through-space interactions between protons. In our case, as mentioned earlier, DTIC interacts mainly with the hydroxyl groups of TN belonging to the cellobiose units. However, because the ROESY experiment was performed in D_2_O, such interactions are not visible, as the protons from the –OH groups were exchanged with deuterium. Nevertheless, weak ROE cross-peaks can still be observed in the region of 3.3–3.8 ppm, corresponding to non-bound protons of TN cellobiose units and DTIC protons. These interactions confirm the non-inclusion geometry of the complex, as the non-bound protons of TN are oriented outward.

**Figure 8:**
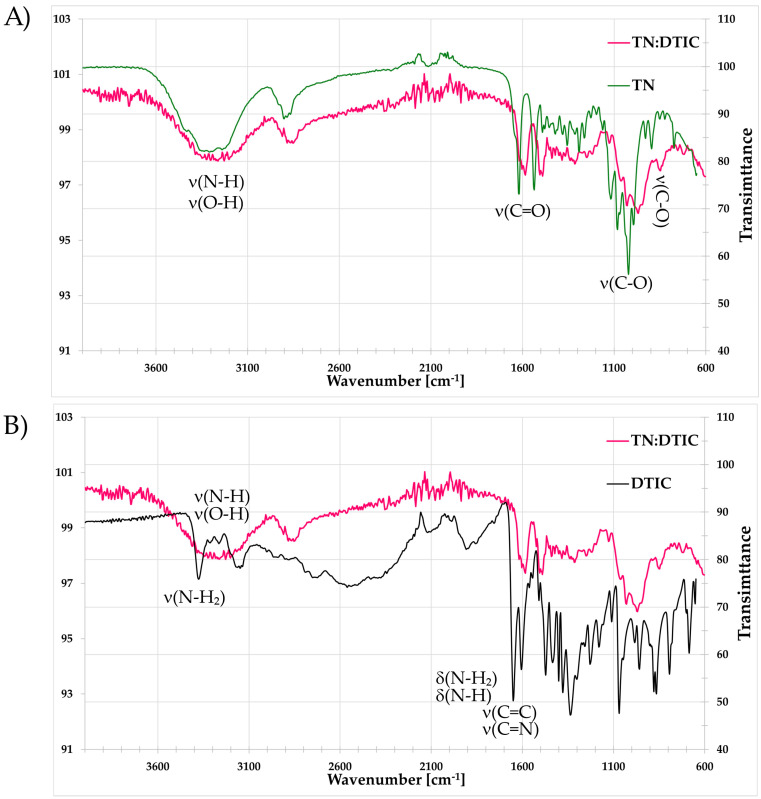
FT-IR spectrum of the complex TN:DTIC (pink line), TN (green line) and DTIC (black line). (**A**) shows absorption bands of the complex and TN, while (**B**) presents absorption bands of the complex and DTIC. Characteristic vibrations are marked: ν—stretching, δ—deformation. For TN:DTIC, an auxiliary transmittance axis is provided on the right to improve readability.

The formation of the complex is confirmed by FT-IR analysis, as illustrated in Figure 8, which shows the spectra of TN:DTIC compared with those of isolated TN and DTIC. The largest changes in the IR absorption bands for the complex compared to TN alone are observed around 1000 cm^−1^ (Δν = 39 cm^−1^), corresponding to C–O stretching vibrations (ν(C–O)), confirming the involvement of the sugar moiety’s hydroxyl groups in complex formation with dacarbazine via hydrogen bonding. Additional band shifts are detected in the 1700–1500 cm^−1^ range, encompassing deformation vibrations of amino groups and stretching vibrations of C=O (urea) from TN, as well as C=C and C=N from dacarbazine.

**Figure 9:**
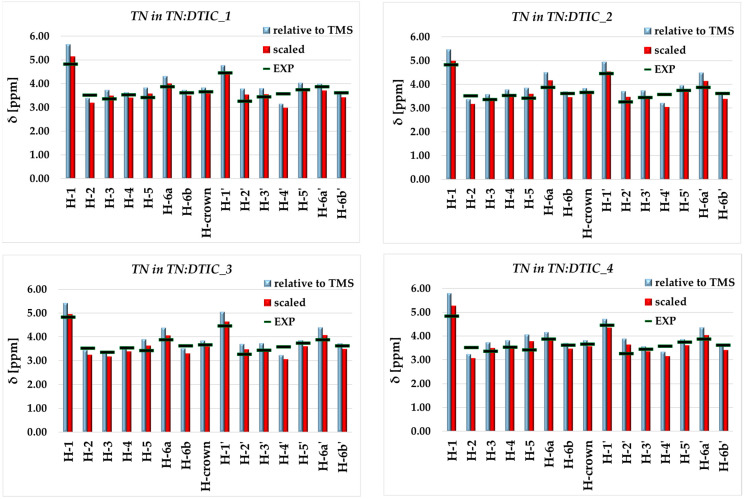
^1^H NMR chemical shifts (δ, ppm) calculated for the most stable complexes (Figure 2) using different theoretical approaches. Experimental values (EXP) were measured in D_2_O. H-6a and H-6b denote the higher and lower chemical shift values of H-6, respectively. The same applies to H-6a′ and H-6b′.

To evaluate the theoretical predictions regarding the most favorable structure of the complex, we compared the experimental NMR results with those obtained from calculations. Figure 9 presents the chemical shifts in TN in each complex, alongside the experimental data. In the theoretical analysis, the chemical shifts were obtained using two approaches: (i) referenced to tetramethylsilane (TMS) and (ii) scaled according to the Tantillo procedure [32]. The chemical shift values obtained for each proton are listed in Tables S5–S8.

As shown, for the two most stable complexes—which are similar in geometry—the calculated chemical shifts exhibit comparable values, although certain differences can be observed. Overall, all the complexes display good agreement with the experimental results, suggesting that in real solution, not only the most stable structure but also other, energetically higher-lying structures may coexist. This is particularly evident for the H-3 proton, for which the best agreement with the experimental data is observed for TN in the TN:DTIC_3 complex.

It is difficult to determine which theoretical approach (Tantillo-scaled or TMS-referenced) provides a more accurate evaluation of the chemical shifts for such a complex system as TN:DTIC. Each method complements the other: in some cases, better agreement with the experimental data is achieved using the scaled approach, while in others, the TMS-referenced method performs better. Nevertheless, it should be emphasized that certain discrepancies between the theoretical and experimental results are to be expected, as the theoretical model cannot fully reproduce the real solution environment.

### 2.3. Thermodynamic Analysis of the Complex

In the dacarbazine system studied with TN, the values of theoretical conductivity Λ_TN(DTIC)_ and the formation constant *K_f_*, were determined in the temperature range of 293.15 to 313.15 K (Table 1).

Data on dacarbazine molar conductivity DTIC in the presence of TN are presented in Table S9.

A clear increase in Λ° was observed with increasing temperature—from 40.52 [S·cm^2^·mol ^−1^] at 293.15 K to 53.30 [S·cm^2^·mol^−1^] at 313.15 K. This phenomenon is typical for electrolyte systems, in which elevated temperature enhances the mobility of ions in the solution and reduces the viscosity of the medium.

At the same time, the values of the formation constant *K_f_* significantly decrease with increasing temperature—from 1.15·10^4^ [dm^3^·mol^−1^] at 293.15 K to 4.29·10^3^ dm^3^·mol^−1^ at 313.15 K. The decrease in *K_f_* indicates a weakening of electrostatic interactions between the ionic forms of dacarbazine and the TN ligand, suggesting partial dissociation of ion pairs at higher temperatures. This change confirms that the process is exothermic in nature.

Increasing the temperature results in a systematic decrease in the association constant $K_{f}$, which confirms the weakening of the stabilizing interactions within the complex at higher molecular kinetic energy. This behavior is consistent with the negative sign of $\Delta H^{0}$ and emphasizes the dominant role of enthalpic factors in stabilizing the formed complex.

The complex formation constant for the dacarbazine and TN system was determined from the measurements of the molar conductivity of the electrolyte solutions. The evaluation was based on a model previously developed and refined in the earlier work of the authors [33,34,35]. The equilibrium relationship between the degree of dissociation and the concentrations of the species is expressed by:

(1) $$K_{f}=\frac{\left(1-\alpha \right)}{\alpha [C_{TN}-\left(1-\alpha \right)C_{DTIC}]}$$

The observed molar conductivity of the solution can be described as

(2) $$\Lambda_{obs}=\alpha \cdot \Lambda_{m}+(1-\alpha ){\cdot \Lambda }_{c}$$

The formation constant of the TN:DTIC complex may also be represented by the following equation:

(3) $$K_{f}=\frac{\left(\Lambda_{m}-\Lambda_{obs}\right)}{\left(\Lambda_{obs}-\Lambda_{c}\right)\cdot C_{TN}}$$

From the above relationship, the concentration of the free TN ligand can be expressed as:

(4) $$\left[C_{TN}\right]=C_{TN}-\frac{C_{TN}(\Lambda_{m}-\Lambda_{obs})}{\left(\Lambda_{m}-\Lambda_{c}\right)}$$

Thus, the overall relationship describing the molar conductivity of the solution during complex formation is given by the following.

(5) $$\Lambda =\left[K_{f}(c_{DTIC}-C_{TN}-1)+\sqrt{K_{f}^{2}(C_{TN}-C_{DTIC}{)}^{2}+2K_{f}\left(C_{DTIC}+C_{TN}\right)+1}\right]\cdot \left[\frac{\Lambda_{DTIC}-\Lambda_{c}}{2K_{f}C_{DTIC}}\right]+\Lambda_{(DTIC)TN}$$

Notations for Equations (4) and (5):

$C_{TN}$—equilibrium concentration of free TN;
$C_{DTIC}$—concentration of dacarbazine in solution;
$\Lambda_{obs}$—observed molar conductivity of the solution in the presence of TN;
$\Lambda_{DTIC}$—molar conductivity of the pure dacarbazine solution before TN addition;
$\Lambda_{(DTIC)TN}$—molar conductivity of the solution containing the TN:DTIC complex;
$\Lambda_{c}$—molar conductivity of the complexed ion;
$K_{f}$—formation constant of the TN:DTIC complex.

The optimal values of $K_{f}$ and $\Lambda_{(DTIC)TN}$ were obtained using a least-squares fitting procedure, by minimizing the sum of squared deviations between the experimental and calculated molar conductivities:

(6) $$\sum_{i=1}^{n}(\Lambda_{exp}-\Lambda_{calc}{)}^{2}$$

where

n—number of experimental points;
$\Lambda_{exp}$—experimentally determined molar conductivity;
$\Lambda_{calc}$—conductivity value calculated from Equation (5).

The limiting molar conductivities of free dacarbazine and its complex with TN were described by empirical relations that account for the effects of the ionic strength.

(7) $$\Lambda_{DTIC}=\Lambda_{0DTIC}-{Sc_{DTIC}}^{\frac{1}{2}}+Ec_{DTIC}lnc_{DTIC}+J_{1}c_{S}+J_{2}c_{DTIC}.^{\frac{3}{2}}$$

(8) $$\Lambda_{(DTIC)TN}=\Lambda_{0(DTIC)TN}-S{c_{DTIC}}^{\frac{1}{2}}+Ec_{DTIC}lnc_{DTIC}+J_{1}c_{S}+J_{2}c_{DTIC}.^{\frac{3}{2}}$$

In these Equations:

$\Lambda_{0}$—limiting molar conductivity at infinite dilution;
$S,E,J_{1},J_{2}$—empirical parameters obtained from data fitting;
$c_{DTIC}$—concentration of dacarbazine;
$c_{S}$—concentration of the supporting electrolyte (background salt) used to correct for ionic strength effects.

Based on the temperature dependence of the formation constant *K_f_*, the standard thermodynamic functions of the association process were calculated: enthalpy (ΔH^0^), Gibbs free energy (ΔG^0^), and entropy (ΔS^0^).

Δ*G*^0^(*T*) = − *R T* ln*K*_A_(*T*) (9)

Δ*G*^0^*(T)* can also be expressed by the polynomial equation:

Δ*G*^0^(*T*) = *A* + *B T* + *C T*^2^ (10)

The entropy, Δ*S*^0^, and enthalpy, Δ*H*^0^, of ion association are defined as:

(11) $$\Delta S^{0}\left(T\right)=-(\frac{\delta \Delta G^{0}}{\delta T}{)}_{p}=-B-2CT$$

Δ*H*^0^ = Δ*G*^0^ + *T* Δ*S*^0^ (12)

The thermodynamic functions described above are (Δ*G*^0^, Δ*S*^0^, Δ*H*^0^) were measured at temperature range T = (293.15–313.15) K and presented in Table 2.

The negative values of ΔG^0^ over the entire temperature range, from −22.79 to −21.78 [kJ·mol^−1^], confirm the spontaneous nature of the complexation process between dacarbazine and TN. At the same time, the ΔH^0^ values are negative, ranging from −42.55 to −32.41 [kJ·mol^−1^], indicating that the association reaction is exothermic. The negative ΔS^0^ values, from −0.0674 to −0.0340 [kJ·mol^−1^·K^−1^], suggest a decrease in system disorder due to the formation of a more organized ionic complex.

All thermodynamic parameters presented in this study were determined exclusively from conductometric measurements, which constituted the principal experimental technique employed. Conductometry enabled direct and quantitative determination of $K_{f}$ and consequently the calculation of $\Delta G^{0}$, $\Delta H^{0}$, and $\Delta S^{0}$ without the need for additional model assumptions.

To gain a more complete understanding of the interactions stabilizing the complex, additional calculations were performed. The theoretical evaluation of the Gibbs free energy is presented in Table S10 for the four most stable complexes. For all analyzed structures, ΔG is negative, which confirms the experimental finding that complex formation is a spontaneous reaction. The best agreement between measurements and calculations is achieved for TN:DTIC_2 and TN:DTIC_4. Again, this may result from the fact that, in the real solution, not only the most stable configurations are present, but also other, higher-energy structures. Therefore, the experimental results may reflect an average effect originating from multiple structures. This tendency was also evident when comparing experimental and theoretical chemical shifts (Figure 9). For some protons, the most stable complex shows the best agreement with the experimental values, while for others, different complexes (TN:DTIC_2 to TN:DTIC_4) match better. We would like to highlight that a similar situation was observed in our recent work [24], where we analyzed the TN complex with treosulfan. In that case, the complex with the lowest stability exhibited the highest agreement with the thermodynamic measurements.

Comparison with previous results for dacarbazine complexes with cyclodextrins [27] shows that the system with TN is characterized by a significantly higher *K_f_* value, indicating stronger interactions between the ligand and dacarbazine. This is most likely due to the presence of numerous donor–acceptor (ureido moieties and sugar units) groups in the TN molecule, which enable hydrogen bonding with nitrogen and oxygen atoms in the drug molecule. Unlike cyclodextrin complexes, where the complexation process is mainly inclusion-based and depends on geometric fitting, in the case of TN, electrostatic interactions and an enthalpy-driven hydrogen-bonding network play a decisive role.

The decrease in *K_f_* values with increasing temperature and the reduction in the absolute value of ΔH^0^ indicate that the TN:DTIC complex is stable at lower temperatures, while partial dissociation occurs at higher temperatures. A similar trend was observed for dacarbazine complexes with α-, HP-β-, and HE-β-cyclodextrins; however, the ΔG^0^ values for TN are more negative, confirming the stronger ionic association.

In summary, the complexation of dacarbazine by TN proceeds spontaneously and is driven mainly by enthalpic factors. The negative ΔH^0^ value reflects the predominance of intermolecular hydrogen-bonding and dipole–dipole interactions, while the negative ΔS^0^ value corresponds to the ordering of molecules within the ionic environment. As the temperature increases, the complex becomes partially destabilized, resulting from the weakening of the hydrogen-bonding network and the increase in molecular kinetic energy.

Moreover, although we did not determine the quantitative solubility of DTIC or the TN:DTIC complex, several experimental observations clearly demonstrate that TN markedly improves the behavior of DTIC in water. The consistently transparent solutions, the absence of precipitation even at high TN:DTIC ratios, together with the high formation constant and favorable thermodynamic parameters, indicate that TN significantly enhances both the effective solubility and the stability of dacarbazine in the aqueous phase.

## 3. Materials and Methods

### 3.1. Computational Analysis

The TN:DTIC complex was analyzed using several computational methods. Initially, we constructed four orientations of the TN:DTIC complex (A, B, C, and D; see Figure S6). It should be noted that we originally generated five orientations, the fifth being denoted as I, corresponding to an inclusion-type structure in which DTIC was placed between the two cellobiose units of TN. However, this geometry became disrupted during optimization (with atoms detaching), and therefore it was not considered in the further analysis and is not included in Figure S6.

All initial configurations were constructed in the HyperChem program *(version 8.0) using the most stable geometries of TN [25] and DTIC [27]. In the latter study, dacarbazine was analyzed, among others, using the M08-HX-D3/6-31G(d,p) method in water (PCM) (a detailed description of this method is provided below). Since TN in ref [25]. had been optimized using a different functional (M06-2X), its geometry was re-optimized at the same level of theory as DTIC. Subsequently, configurational sampling was started for each initial arrangement (A; B; C and D). A graphical representation of the theoretical workflow applied in this study is shown in Chart S1.

First, a global optimization procedure was carried out using GOAT (Global Optimizer Algorithm) as implemented in ORCA 6.0.1 [36]. These optimizations were performed at the xTB level of theory [37]. This stage enabled broad sampling of the conformational landscape and ensured that the starting geometries for further refinement were not biased by the initial arrangement of the two molecules.

Next, each of the structures obtained from the GOAT optimization was transferred to the HyperChem program [38], where numerous additional configurations were generated. In this step, DTIC was systematically rotated around TN along the X, Y, and Z axes in 20° increments, generating multiple orientations for each initial complex. This procedure yielded 1470 structures for each configuration.

Subsequently, all complexes generated in HyperChem were re-optimized in the gas phase using the semi-empirical PM7 method [39] implemented in MOPAC2016 [40]. Based on the PM7 heat of formation values, ten of the most stable structures from each configuration (A; B; C AMD D) were selected for higher-level quantum-chemical refinement.

The final optimization stage was performed at the density functional theory (DFT) level using the M08-HX-D3/6-31G(d,p) method. In this approach, M08-HX denotes the Minnesota 2008 high-X functional [41], D3 represents Grimme’s empirical dispersion correction [42], and 6-31G(d,p) is the Pople basis set [43]. All DFT optimizations were performed in aqueous solution using the PCM implicit solvent model. These calculations were carried out with Gaussian 16 (Revision C.02) [44].

For all structures optimized at the DFT level, harmonic vibrational frequency analyses were performed to confirm that the stationary points correspond to true minima on the potential energy surface. The absence of imaginary frequencies verified the structural stability of the complexes.

The energetic stability of the complexes was estimated by calculating the complexation (E_compl) energy using following equations:

(13) $$E_{\_compl}=E_{complex}^{OPT}-\left(E_{TN}^{OPT}+E_{DTIC}^{OPT}\right)$$

where

${\;E}_{complex}^{OPT}$—is the energy of the optimized complex;
$E_{TN}^{OPT}$ and ${\;E}_{DTIC}^{OPT}$—are the energies of TN and DTIC, respectively, in their most stable geometries.

The complexation energies were further corrected for basis set superposition error (BSSE) using the counterpoise (CP) correction method [45].

NMR parameters were evaluated using the Gauge-Including Atomic Orbital (GIAO) formalism [46]. For all geometries previously optimized at the M08-HX-D3/6-31G(d,p) level of theory, isotropic magnetic shielding constants for ^1^H nuclei were computed in aqueous solution employing the PCM solvation model in combination with the M08-HX/6-31++G(d,p) method. Chemical shifts (δ) were then obtained using two approaches: (i) with reference to tetramethylsilane (TMS), and (ii) following the Tantillo procedure [32], where δ is calculated as a linear function of the isotropic shielding constant. For nuclei occupying symmetry-equivalent positions within the two cellobiosyl moieties, the final δ values were reported as the arithmetic mean of the corresponding scaled shifts.

### 3.2. Synthesis of TN:DTIC Complex

A detailed synthetic procedure for the preparation of cryptand TN, together with its NMR characterization, has been reported previously [47]. Dacarbazine was obtained from Sigma-Aldrich and used without further purification. For the preparation of the TN:DTIC mixture, equimolar amounts of TN and DTIC were dissolved in 1 mL of water, and the solution was stirred for 24 h under the argon atmosphere to ensure complete interaction between the compounds. Subsequently, the samples were transferred to D_2_O, and the NMR spectra were recorded.

### 3.3. Thermochemistry Analysis

Solutions were prepared gravimetrically using a Sartorius RC 210D analytical balance with an accuracy of ±1·10^−5^ g. Electrical conductivity measurements were carried out according to the procedure previously described by Bešter-Rogač and co-workers [27,48,49,50]. A three-electrode measurement cell made of PYREX glass, connected to a Precise Component Analyzer 6430B bridge (Wayne-Kerr, London, UK), was used. Measurements were performed within the frequency range ν = 0.2–20 kHz (0.2, 0.5, 1, 2, 3, 5, 10, and 20 kHz). The solution temperature was stabilized with an accuracy of ±0.003 K using an UltraUB 20F thermostat with a DLK 25 flow cooler (Lauda, Lauda-Königshofen, Germany). A detailed description of the conductometric measurement procedure is given in the literature [50,51].

The conductometric cell was calibrated at each temperature using aqueous potassium chloride (KCl) solutions [52]. The specific conductivity λ = 1/R∞ was calculated by extrapolating the cell resistance dependence R(ν) to infinite frequency according to the empirical equation R(ν) = R∞ + A/ν. All obtained values were corrected for the specific conductivity of the pure solvent.

## 4. Conclusions

The present study provides a comprehensive analysis of the complexation between dacarbazine (DTIC) and 1,10-*N,N′*-bis-(β-D-ureidocellobiosyl)-4,7,13,16-tetraoxa-1,10-diazacyclooctadecane (TN) using a combination of theoretical, spectroscopic, and thermochemical approaches. Theoretical calculations revealed that TN predominantly forms non-inclusion complexes, in which DTIC interacts mainly with the sugar units through hydrogen bonding. The formation of an inclusion complex appears to be unlikely due to the highly compact geometry of TN, stabilized by numerous intramolecular hydrogen bonds.

Spectroscopic measurements (^1^H NMR) performed in heavy water confirmed the formation of the TN:DTIC complex. The most pronounced chemical shift changes were observed for the protons associated with the sugar units and the diazacrown ether moiety of TN. Comparison with theoretical predictions indicates that the M08-HX-D3 method accurately captures the most energetically favorable configurations of the complex. Moreover, DFT results suggest that in real solution, not only the most stable conformers but also other higher-energy configurations may coexist, resulting in a dynamic equilibrium of multiple complex geometries.

Two theoretical approaches for calculating NMR chemical shifts were examined—the standard TMS-referenced method and the scaling approach proposed by the Tantillo group. Both methods showed good agreement with experimental data. Thermodynamic analysis further confirmed that the TN:DTIC complex is thermodynamically stable even at elevated temperatures (up to 313 K). The complex formation process is spontaneous, exothermic, and enthalpy-driven, as evidenced by the negative values of ΔG^0^, ΔH^0^, and ΔS^0^.

As a final remark, we would like to emphasize that our study not only advances our fundamental understanding of TN:DTIC complexation but also suggests several potential societal and practical implications. By elucidating the molecular interactions and stability of this complex, our findings contribute to the rational design of TN-based drug delivery systems that may enhance the stability and bioavailability of photosensitive anticancer drugs like dacarbazine. These aspects may be of potential clinical relevance, as improved drug stability and targeted delivery could help to reduce side effects and increase therapeutic efficacy, thereby potentially benefiting patient care.

With a view toward future perspectives, further studies are planned to explore the pharmaceutical potential of TN-based systems, including drug-release behavior, cellular uptake, and selected biological activities. Additional preclinical studies will be required to clarify the safety and therapeutic potential of TN-based drug delivery platforms.

## Figures and Tables

**Table 1:** The value of constant formation *K_f_* [dm^3^/mol] and theoretical conductivity Λ_TN(DTCI)_ [S∙cm^2^/mol^−1^].

T/K	Λ_TN(DTIC)_ [S∙cm^2^/mol^−1^]	*K*_f/_dm^3^·mol^−1^	σ(Λ)
293.15	40.52 ± 0.01	11,526.9 ± 2	0.02
298.15	44.85 ± 0.01	8657.4 ± 2	0.01
303.15	47.90 ± 0.02	6717.8 ± 3	0.01
308.15	50.75 ± 0.01	5287.4 ± 2	0.01
313.15	53.30 ± 0.01	4293.3 ± 2	0.02

**Table 2:** Standard thermodynamic quantities for the tested compounds in the temperature range from T = (293.15 to 313.15) K.

T/K	Δ*H*^0^/kJ·mol^−1^	Δ*G*^0^/kJ·mol^−1^	Δ*S*^0^/kJ·mol^−1^·K^−1^
293.15	−42.55	−22.79	−0.0674
298.15	−40.07	−22.47	−0.0590
303.15	−37.57	−22.21	−0.0507
308.15	−35.00	−21.96	−0.0423
313.15	−32.41	−21.78	−0.0340

## Data Availability

The original contributions presented in this study are included in the article/Supplementary Materials. Further inquiries can be directed to the corresponding author.

## Supplementary Materials

The following supporting information can be downloaded at https://www.mdpi.com/article/10.3390/molecules30244819/s1, Figure S1: ^1^H NMR spectrum of DTIC measured in D_2_O; Figure S2: ^1^H NMR spectrum of TN measured in D_2_O; Figure S3: The COSY spectrum of DTIC; Figure S4: The COSY spectrum of TN; Figure S5: The COSY spectrum of the TN:DTIC complex; Figure S6: Initial model of the complexes considered during the configurational search; Table S1: Total energy values obtained at the M08-HX-D3/6-31G(d,p) level of theory in water (PCM) for the most stable complexes presented in Figure 2 of the main article; Table S2: Coordinates [Å] of the most stable complexes, presented in Figure 2 of the main article, obtained at the M08-HX-D3/6-31G(d,p) level of theory in water (PCM); Table S3: The geometrical parameters of hydrogen bonds X–H···A formed in the most stable complexes of TN:DTIC; Table S4: Chemical shifts of TN and DTIC protons in the free state and in the complex, along with the corresponding complexation-induced changes (Δδ); Table S5: ^1^H NMR chemical shifts values of TN in the TN:DTIC_1 complex obtained at the M08-HX/6-31++G(d,p) level of theory in water (PCM); Table S6: ^1^H NMR chemical shifts values of TN in the TN:DTIC_2 complex obtained at the M08-HX/6-31++G(d,p) level of theory in water (PCM); Table S7: ^1^H NMR chemical shifts values of TN in the TN:DTIC_3 complex obtained at the M08-HX/6-31++G(d,p) level of theory in water (PCM); Table S8: ^1^H NMR chemical shifts values of TN in the TN:DTIC_4 complex obtained at the M08-HX/6-31++G(d,p) level of theory in water (PCM); Table S9: The value of molar concentration of salt C_DTIC_ [mol/dm^3^], concentration of ligand C_TN_ [mol/dm^3^], molar conductivity Λ_m_ [S‧cm^2^‧mol^−1^] for TN with DTIC in water at all tested temperatures at pressure *p* = 0.1 MPa; Table S10: Theoretical values of the Gibbs free energy (ΔG) [kJ/mol] obtained for the most stable complexes at the M08-HX-D3/6-31G(d,p) level of theory in water (PCM); Chart S1: Flowchart illustrating the computational analysis carried out in this study.
