# Supplementary material for: Exploring Dacarbazine Complexation with a Cellobiose-Based Carrier: A Multimethod Theoretical, NMR, and Thermochemical Study
Source: Molecules. 2025 Dec 18;30(24):4819. doi: 10.3390/molecules30244819 (PMC12735980; doi:10.3390/molecules30244819)
Supplement: Supplementary file 1 [file molecules-30-04819-s001.zip › molecules-4017318-supplementary.pdf]

Electronic Supplementary File

Exploring Dacarbazine Complexation with a Cellobiose-Based Carrier: A Multimethod Theoretical, NMR, and Thermochemical Study

Marta Hoelm[a]*, Zdzisław Kinart[a] and Stanisław Porwański[b]

[a] University of Lodz, Faculty of Chemistry, Department of Physical Chemistry, Pomorska 163/165, Lodz, 90-236, Poland; marta.hoelm@chemia.uni.lodz.pl; zdzislaw.kinart@chemia.uni.lodz.pl;
[b] University of Lodz, Faculty of Chemistry, Department of Organic and Applied Chemistry, Tamka 12, 91-403 Lodz, Poland; stanislaw.porwanski@chemia.uni.lodz.pl

* Correspondence: marta.hoelm@chemia.uni.lodz.pl;

Table S1. Total energy values [Hartree] obtained at the M08-HX-D3/6-31G(d,p) level of theory in water (PCM) for the most stable complexes presented in Figure 2 of the main article.

Molecule	Energy
TN:DTIC_1	-4300.903018
TN:DTIC_2	-4300.901228
TN:DTIC_3	-4300.903105
TN:DTIC_4	-4300.900822

Table S2. Coordinates [Å] of the most stable complexes, presented in Figure 2 of the main article, obtained at the M08-HX-D3/6-31G(d,p) level of theory in water (PCM).

Atoms	TN:DTIC_1			TN:DTIC_2			TN:DTIC_3			TN:DTIC_4		
	x	y	z	x	y	z	x	y	z	x	y	z
C	4.18171	-3.82010	-3.19266	3.90158	-4.27573	-2.76973	4.49570	1.97072	-3.62150	-3.90985	-3.53344	3.54703
C	4.44056	-4.48088	-1.84050	4.08481	-4.82698	-1.35753	4.77164	0.52349	-4.02704	-4.23813	-4.31948	2.27994
O	3.23081	-4.46991	-1.09501	2.87055	-4.64867	-0.64041	3.56201	-0.21025	-3.92111	-3.05923	-4.41070	1.49235
C	3.34325	-4.06977	0.25714	2.99741	-4.11515	0.66392	3.66180	-1.48506	-3.31277	-3.21696	-4.14752	0.11127
C	3.53648	-2.55866	0.37891	3.32031	-2.62124	0.63394	3.78116	-1.35307	-1.79540	-3.39719	-2.65316	-0.15077
O	3.56276	-2.16656	1.74669	3.38756	-2.10338	1.95674	3.69987	-2.62135	-1.15558	-3.44936	-2.38893	-1.54900
C	4.82285	-2.29319	2.41020	4.64301	-2.25291	2.62350	4.91073	-3.37278	-1.07819	-4.71390	-2.60125	-2.17981
C	4.83086	-1.34048	3.60534	4.72844	-1.19519	3.72418	4.76832	-4.35611	0.08099	-4.75349	-1.76673	-3.45939
H	3.30064	-4.27738	-3.66968	2.98701	-4.69497	-3.21781	3.65465	2.37601	-4.20703	-3.02795	-3.97029	4.04100
H	5.02906	-4.01846	-3.86237	4.73485	-4.61249	-3.40061	5.36595	2.59228	-3.86976	-4.73853	-3.63162	4.26107
H	4.77330	-5.52089	-1.98806	4.32971	-5.90017	-1.40315	5.14189	0.48826	-5.06458	-4.58599	-5.33138	2.54357
H	5.23666	-3.93537	-1.30638	4.91697	-4.30290	-0.85818	5.54847	0.09904	-3.36867	-5.04382	-3.80891	1.72608
H	4.16591	-4.61052	0.75805	3.76233	-4.66711	1.23864	4.50874	-2.05604	-3.73309	-4.06356	-4.72484	-0.30138
H	2.40052	-4.36007	0.74468	2.02600	-4.27177	1.15659	2.73362	-2.02045	-3.56106	-2.29612	-4.49742	-0.37826
H	2.67605	-2.03844	-0.06703	2.50397	-2.07623	0.13716	2.92487	-0.77814	-1.41816	-2.52249	-2.10446	0.22723
H	4.44978	-2.22214	-0.13945	4.25673	-2.41729	0.08782	4.70657	-0.83154	-1.50213	-4.29708	-2.26007	0.34984
H	5.63971	-2.03262	1.71367	5.46921	-2.11577	1.90303	5.75977	-2.69144	-0.89010	-5.52666	-2.28794	-1.50017
H	4.96417	-3.32880	2.75804	4.71761	-3.25872	3.06667	5.08385	-3.91469	-2.02164	-4.84139	-3.66774	-2.42401
H	5.59122	-1.68269	4.31881	5.46211	-1.52613	4.47010	5.48222	-5.17829	-0.05906	-5.52077	-2.18310	-4.12472
H	3.86294	-1.41156	4.12433	3.75867	-1.14339	4.24232	3.76437	-4.80560	0.04768	-3.79391	-1.87381	-3.98731
C	-5.34861	1.93622	-1.31700	-5.30827	1.95594	-1.36046	-5.18719	1.61851	1.40930	4.99671	2.10097	1.66614
C	-5.55937	1.60724	0.15473	-5.44247	1.66706	0.12809	-5.49464	0.12815	1.48962	5.42214	1.78553	0.23568
C	-4.42686	2.15922	1.00239	-4.31594	2.30208	0.91916	-4.48857	-0.59676	2.36882	4.39381	2.23521	-0.78800
C	-3.12227	1.57956	0.46603	-2.99865	1.78751	0.34991	-3.10316	-0.31865	1.79674	3.07424	1.57105	-0.41800
O	-2.96971	1.92645	-0.90604	-2.91382	2.10015	-1.03541	-2.87254	1.08471	1.76596	2.72524	1.94348	0.91076
C	-3.99218	1.35666	-1.72782	-3.93144	1.44083	-1.79704	-3.75729	1.76773	0.87917	3.63314	1.44462	1.89253
O	-4.56563	1.75008	2.34699	-4.38032	1.91456	2.27684	-4.70725	-1.99138	2.32585	4.75742	1.79810	-2.08123
C	-3.65286	1.62469	-3.18032	-3.65736	1.63341	-3.27583	-3.32586	3.21643	0.81072	3.02353	1.73929	3.24921
O	-2.56819	0.82504	-3.59682	-2.63227	0.77144	-3.72746	-1.97583	3.29693	0.39222	1.77778	1.06944	3.36659
O	-6.42016	1.43153	-2.08965	-6.36895	1.33917	-2.06068	-6.15346	2.28278	0.62216	5.98866	1.65664	2.56898
O	-6.78070	2.10482	0.65604	-6.67015	2.10673	0.66409	-6.79300	-0.12731	1.98028	6.67214	2.35377	-0.09042
O	-2.05878	2.11870	1.17095	-1.94568	2.40716	1.00434	-2.13490	-0.89588	2.60050	2.07910	2.01016	-1.27041
H	-5.33760	3.03143	-1.45349	-5.37853	3.04086	-1.54708	-5.23993	2.05793	2.42046	4.90016	3.19196	1.79882
H	-5.52239	0.50258	0.25125	-5.34538	0.56562	0.23410	-5.37789	-0.28989	0.46960	5.46642	0.68208	0.15073
H	-4.38936	3.26202	0.91706	-4.34571	3.40291	0.81059	-4.53555	-0.21006	3.40443	4.26258	3.33312	-0.75142
H	-3.14412	0.47474	0.56029	-2.95107	0.68546	0.47855	-3.05320	-0.71929	0.76162	3.18725	0.46688	-0.46851
H	-4.01198	0.25811	-1.56287	-3.88699	0.35626	-1.57507	-3.70023	1.30515	-0.12875	3.74920	0.34582	1.76695
H	-5.49415	1.87386	2.58368	-5.31160	1.94161	2.53376	-5.65545	-2.13254	2.44493	5.68031	2.04989	-2.21532
H	-3.43264	2.69506	-3.30979	-3.40486	2.68716	-3.46824	-3.43329	3.67391	1.80648	2.86978	2.82606	3.33783
H	-4.53527	1.37278	-3.78859	-4.58098	1.38514	-3.81926	-3.99921	3.74796	0.11754	3.71914	1.40436	4.03362
H	-1.74192	1.34274	-3.60773	-1.76767	1.22303	-3.71620	-1.57679	4.06306	0.84215	1.07709	1.74774	3.45072
H	-6.27457	0.47984	-2.24955	-6.26128	0.37648	-1.94265	-5.98108	2.09524	-0.32088	6.00474	0.67935	2.55271
H	-7.45782	1.88044	0.00355	-7.35635	1.80327	0.05472	-7.37742	0.51688	1.55755	7.23182	2.24480	0.69092

C	-0.94713	1.25758	1.39709	-0.83610	1.58176	1.32643	-0.96468	-1.35211	1.92704	0.98741	1.11304	-1.45911
C	-0.50706	1.49544	2.84210	-0.33609	2.01061	2.70167	-0.64154	-2.73275	2.50328	0.55723	1.29489	-2.91835
O	0.75236	0.90607	3.11434	0.86955	1.33969	3.01939	0.61603	-3.21117	2.05983	-0.68335	0.67244	-3.20505
C	1.82662	1.21186	2.23189	1.93297	1.42827	2.07679	1.74188	-2.35648	2.21030	-1.78332	0.95748	-2.34646
C	1.44001	0.79333	0.80911	1.47829	0.89447	0.71037	1.47300	-1.01062	1.52420	-1.39262	0.60313	-0.91236
C	0.16489	1.52202	0.39238	0.24247	1.66577	0.25720	0.18928	-0.36252	2.05110	-0.15189	1.38081	-0.48486
O	-0.18923	1.06010	-0.89435	-0.20935	1.09421	-0.95338	-0.03767	0.79744	1.27497	0.13375	0.94751	0.82670
O	2.51690	1.05636	-0.05658	2.55555	1.01185	-0.18966	2.59861	-0.18805	1.70645	-2.47776	0.83933	-0.05162
C	-1.47023	0.77769	3.78234	-1.32245	1.55203	3.77053	-1.62936	-3.75136	1.93930	1.55155	0.56630	-3.81785
O	-1.41223	-0.62132	3.54091	-1.44834	0.13546	3.73619	-1.43121	-3.85404	0.53606	1.49138	-0.82710	-3.54804
H	-1.27731	0.20726	1.31023	-1.17273	0.53376	1.41707	-1.20364	-1.49195	0.85903	1.35182	0.07803	-1.33750
H	-0.49158	2.58277	3.04355	-0.20627	3.10894	2.72900	-0.69266	-2.69341	3.60701	0.52507	2.37578	-3.15155
H	2.08817	2.28672	2.26430	2.29127	2.47077	1.97382	1.99194	-2.21011	3.27528	-2.09749	2.01395	-2.42441
H	1.24094	-0.29609	0.86460	1.20189	-0.16807	0.85894	1.33555	-1.24318	0.44824	-1.14337	-0.47769	-0.93269
H	0.37786	2.60803	0.37684	0.52220	2.72636	0.11331	0.33854	-0.09825	3.11730	-0.37957	2.46574	-0.50708
H	-1.00176	1.51253	-1.18446	-0.97190	1.60234	-1.28284	-0.86460	1.23334	1.54354	0.92050	1.40681	1.16517
H	2.37326	0.59401	-0.89944	2.41655	0.42287	-0.94968	2.62529	0.44815	0.97275	-2.24243	0.47879	0.82268
H	-1.20802	1.00603	4.82796	-0.97046	1.88620	4.75966	-1.47985	-4.72657	2.43009	1.32315	0.77233	-4.87579
H	-2.49849	1.11297	3.58269	-2.31434	1.97794	3.56955	-2.66044	-3.40986	2.11377	2.57173	0.91280	-3.59280
H	-0.48428	-0.88220	3.40384	-0.56681	-0.24902	3.58780	-0.47380	-3.85039	0.35872	0.56075	-1.07899	-3.41310
C	-6.72375	-1.87334	1.45353	-6.71217	-1.88474	1.39937	-6.52769	-2.20113	-1.44668	6.79940	-1.42387	-1.46479
C	-5.76479	-2.37541	2.52333	-5.86145	-1.97095	2.65067	-5.60624	-3.40186	-1.60460	5.95170	-2.05595	-2.56025
C	-4.35410	-1.85072	2.27671	-4.46095	-1.52824	2.27620	-4.19986	-3.07357	-1.11602	4.47411	-1.75383	-2.33920
C	-3.93485	-2.27487	0.87024	-3.89586	-2.44319	1.17745	-3.70461	-1.84344	-1.87301	4.08977	-2.22192	-0.93764
O	-4.85294	-1.72550	-0.05428	-4.83016	-3.24833	0.50745	-4.60192	-0.77680	-1.63609	4.90622	-1.54450	-0.00070
C	-6.17340	-2.23073	0.07289	-6.20802	-2.88846	0.35622	-5.90544	-0.99847	-2.15448	6.28041	-1.88986	-0.10588
O	-3.52305	-2.39330	3.27330	-3.66397	-1.56038	3.44202	-3.39690	-4.20372	-1.34834	3.74612	-2.41529	-3.34388
C	-6.96101	-1.57059	-1.06007	-6.46256	-2.41840	-1.06812	-6.66925	0.29963	-1.89235	6.98221	-1.20102	1.06894
O	-6.15487	-1.39317	-2.21739	-5.68261	-1.27888	-1.36986	-5.82335	1.43162	-2.03549	6.13571	-1.13602	2.20669
O	-7.99876	-2.45794	1.58161	-8.06415	-2.20316	1.65504	-7.79486	-2.43905	-2.01348	8.15350	-1.79529	-1.57455
O	-6.25999	-1.95464	3.77423	-6.41589	-1.10417	3.61510	-6.16607	-4.47292	-0.87869	6.39994	-1.55247	-3.79872
O	-2.69215	-1.74888	0.59181	-3.16982	-1.62175	0.28747	-2.47461	-1.48244	-1.36583	2.77971	-1.85966	-0.71097
H	-6.78090	-0.76663	1.53657	-6.63196	-0.84972	1.00943	-6.60903	-1.96775	-0.36286	6.67420	-0.32110	-1.52436
H	-5.73447	-3.48401	2.46973	-5.84180	-3.01681	3.01735	-5.54632	-3.65303	-2.68436	6.09143	-3.15649	-2.51464
H	-4.36387	-0.74183	2.30824	-4.52488	-0.49684	1.88847	-4.23913	-2.80695	-0.03909	4.32216	-0.65518	-2.37021
H	-3.93128	-3.38274	0.78272	-3.21270	-3.16758	1.65337	-3.64617	-2.04940	-2.96361	4.22929	-3.31930	-0.83016
H	-6.16210	-3.33211	-0.04148	-6.77489	-3.81718	0.53280	-5.84181	-1.20448	-3.24075	6.38920	-2.98952	-0.02948
H	-2.77660	-1.78223	3.41244	-3.00796	-0.84149	3.43351	-2.66529	-4.17938	-0.70484	2.91340	-1.92654	-3.47234
H	-7.32540	-0.58487	-0.71521	-7.52300	-2.14301	-1.16546	-7.08120	0.27358	-0.86600	7.27952	-0.17900	0.76944
H	-7.84166	-2.17637	-1.31194	-6.25324	-3.23604	-1.77518	-7.51871	0.38289	-2.58328	7.90313	-1.74478	1.32090
H	-5.22919	-1.59372	-1.98506	-4.77013	-1.55530	-1.57600	-4.89828	1.12691	-2.10041	5.24439	-1.45033	1.96039
H	-8.27071	-2.33717	2.50027	-8.35364	-1.61538	2.36430	-8.10856	-3.27901	-1.65503	8.41851	-1.62104	-2.48656
H	-5.58480	-2.18662	4.42528	-5.74285	-0.99960	4.30092	-5.51375	-5.18536	-0.90009	5.77553	-1.87178	-4.46331
C	-1.87143	-2.40538	-0.37188	-2.10815	-2.26771	-0.40410	-1.60034	-0.70575	-2.17790	2.03485	-2.54395	0.29196
C	-0.47049	-2.29972	0.24745	-0.77379	-1.99500	0.31492	-0.21500	-1.20384	-1.74890	0.59504	-2.38963	-0.21812
O	0.53861	-2.80011	-0.60918	0.29530	-2.61745	-0.38593	0.83899	-0.44103	-2.30569	-0.37217	-2.81898	0.72202
C	0.64781	-2.01730	-1.79164	0.50403	-2.03750	-1.67169	0.82040	0.91736	-1.85574	-0.34738	-2.02321	1.91323
C	-0.66530	-2.09995	-2.57449	-0.76275	-2.17767	-2.52558	-0.47272	1.54764	-2.38427	1.00146	-2.28489	2.59122
C	-1.84777	-1.64301	-1.69811	-1.97140	-1.61081	-1.77227	-1.68876	0.77964	-1.84663	2.13359	-1.85323	1.64786
O	-3.05355	-1.77911	-2.42562	-3.15031	-1.77315	-2.53341	-2.86645	1.36919	-2.36864	3.37333	-2.10209	2.28801
O	-0.59381	-1.35567	-3.76361	-0.60185	-1.56462	-3.77792	-0.60928	2.90968	-2.05635	1.16392	-1.62827	3.82663
C	-0.30844	-2.94430	1.61532	-0.67297	-2.39587	1.77749	0.01959	-2.67612	-2.04974	0.35348	-3.09007	-1.54794
O	0.65033	-2.24673	2.40111	0.40640	-1.72845	2.41813	0.90096	-3.26654	-1.10790	-0.62617	-2.42748	-2.33271
H	-2.19636	-3.45188	-0.51105	-2.31233	-3.34851	-0.50623	-1.80104	-0.88305	-3.24987	2.35226	-3.60044	0.36118
H	-0.31891	-1.21537	0.37973	-0.63679	-0.90210	0.26187	-0.20229	-1.08061	-0.65385	0.47589	-1.30587	-0.37601
H	0.83968	-0.95750	-1.54208	0.74536	-0.96173	-1.58116	0.84507	0.97062	-0.75332	-0.44177	-0.95181	1.66934
H	-0.83320	-3.15197	-2.86389	-0.94249	-3.25088	-2.71201	-0.46141	1.48012	-3.48608	1.08268	-3.36792	2.78879
H	-1.68908	-0.57807	-1.43779	-1.79265	-0.53035	-1.59734	-1.66846	0.85850	-0.73597	2.00196	-0.76598	1.45279
H	-2.96706	-1.11156	-3.12946	-3.07643	-1.10089	-3.23546	-2.69888	2.32378	-2.36177	3.20488	-1.95062	3.23057
H	-0.33604	-0.43735	-3.56659	-0.32226	-0.63782	-3.66340	-0.84771	2.98636	-1.11312	1.27404	-0.67080	3.66589
H	-1.26233	-2.88663	2.15590	-1.57857	-2.07192	2.30699	-0.94007	-3.20591	-1.97149	1.28817	-3.06868	-2.12567

H	-0.03993	-4.00664	1.49405	-0.58892	-3.49156	1.87031	0.39100	-2.78364	-3.08251	0.08406	-4.14314	-1.36151
H	1.55275	-2.50886	2.17001	1.23981	-2.19798	2.27632	1.82336	-3.13125	-1.37095	-1.51951	-2.68286	-2.05978
N	4.02725	-2.37213	-3.10196	3.87886	-2.81772	-2.82141	4.25100	2.11264	-2.18982	-3.71181	-2.10971	3.29826
N	5.11827	0.05654	3.29424	5.12141	0.14094	3.28706	5.00480	-3.78197	1.40035	-5.05400	-0.35063	-3.27241
C	5.15286	-1.55882	-3.54813	5.07386	-2.15806	-3.33505	5.33073	2.67347	-1.38080	-4.79124	-1.21789	3.70581
C	6.36398	-1.64201	-2.61884	6.26302	-2.24211	-2.37843	6.53304	1.74309	-1.22029	-6.05914	-1.37354	2.86476
O	6.07272	-1.27112	-1.29077	5.98894	-1.70082	-1.10645	6.20718	0.50396	-0.63469	-5.84790	-1.17326	1.48550
C	5.78116	0.11146	-1.15124	5.80935	-0.29222	-1.13105	5.88812	0.60217	0.74490	-5.50769	0.16555	1.16312
C	5.60647	0.42283	0.31425	5.64754	0.19455	0.28677	5.61859	-0.78254	1.28298	-5.41778	0.29922	-0.33786
O	6.82377	0.19168	1.00406	6.83845	-0.05503	1.01444	6.80863	-1.55338	1.23989	-6.67783	0.00759	-0.92151
C	7.05243	1.06972	2.08948	7.12441	0.90259	2.01560	6.94011	-2.48365	2.29800	-6.96494	0.74867	-2.09082
C	6.49108	0.53332	3.40811	6.52425	0.52714	3.37205	6.35083	-3.85372	1.95587	-6.44174	0.08557	-3.36810
C	2.88971	-1.75217	-2.68705	2.79672	-2.06148	-2.49404	3.08960	1.75581	-1.57851	-2.55628	-1.56950	2.81572
C	4.15100	0.98124	2.99713	4.22790	1.09892	2.88359	4.01585	-3.27963	2.20729	-4.09391	0.61208	-3.09465
H	4.80162	-0.52643	-3.64098	4.81472	-1.11619	-3.54746	4.91143	2.94240	-0.40604	-4.41054	-0.19304	3.65721
H	5.45748	-1.89789	-4.55052	5.35536	-2.63168	-4.28836	5.67236	3.60355	-1.86105	-5.04699	-1.42725	4.75683
H	7.16953	-1.00908	-3.03413	7.12650	-1.73213	-2.84347	7.30352	2.26751	-0.62607	-6.82110	-0.67080	3.24907
H	6.74612	-2.67312	-2.57728	6.55049	-3.29176	-2.21503	6.97545	1.51825	-2.20249	-6.46800	-2.38980	2.96983
H	4.84365	0.37090	-1.67256	4.90096	-0.02063	-1.69524	4.98324	1.21398	0.89475	-4.52226	0.42797	1.58847
H	6.60829	0.71027	-1.57567	6.68667	0.18610	-1.60476	6.73019	1.06719	1.28997	-6.27181	0.85287	1.57156
H	4.78399	-0.18871	0.72166	4.77621	-0.30477	0.74401	4.81033	-1.25152	0.69600	-4.63268	-0.37356	-0.71504
H	5.29147	1.47437	0.42343	5.41600	1.27350	0.27577	5.24143	-0.69992	2.31667	-5.08333	1.32301	-0.58796
H	6.61983	2.06035	1.87448	6.76117	1.89622	1.70681	6.46709	-2.09036	3.21119	-6.55432	1.76563	-2.00224
H	8.14068	1.18932	2.19547	8.21808	0.95657	2.11959	8.01489	-2.60846	2.49640	-8.05905	0.83054	-2.16758
H	6.56858	1.31835	4.17669	6.65609	1.36932	4.06946	6.37004	-4.48638	2.85763	-6.57499	0.78330	-4.21006
H	7.09780	-0.31961	3.73600	7.06786	-0.33459	3.77835	6.98140	-4.33274	1.19647	-7.04076	-0.80938	-3.57721
N	1.77150	-2.52872	-2.52420	1.62124	-2.72031	-2.25626	1.99053	1.55669	-2.37408	-1.47245	-2.41356	2.70906
N	2.93153	0.44262	2.68793	2.97955	0.62189	2.59632	2.82828	-3.02207	1.56934	-2.85133	0.12149	-2.78279
O	4.38227	2.19285	2.99470	4.54666	2.28572	2.77169	4.20128	-3.06435	3.40486	-4.34416	1.81500	-3.19231
O	2.84535	-0.52116	-2.51030	2.85654	-0.81815	-2.45852	3.01431	1.67894	-0.33870	-2.46476	-0.36089	2.54497
H	1.91967	-3.51980	-2.36446	1.68070	-3.69504	-1.97948	2.16936	1.33093	-3.34594	-1.67832	-3.40436	2.63166
H	2.92083	-0.52841	2.38940	2.89083	-0.37221	2.40668	2.87406	-2.89215	0.56350	-2.82789	-0.81314	-2.38593
C	-1.53836	4.69504	-2.03766	-1.30643	4.67993	-2.39717	1.90415	2.44657	3.11549	-1.44412	2.28368	3.33327
N	-1.21690	3.73433	-2.86831	-1.06832	3.62417	-3.13529	0.86510	2.87478	3.79188	-0.29827	2.91860	3.25281
C	0.10029	3.44677	-2.60689	0.23795	3.28388	-2.87987	0.20894	3.73384	2.94052	-0.37021	3.68686	2.11247
C	0.56975	4.25337	-1.58155	0.78610	4.15732	-1.95271	0.88896	3.81895	1.73471	-1.60771	3.50503	1.51387
N	-0.49177	5.04093	-1.23624	-0.21631	5.03875	-1.66186	1.97155	3.00480	1.87848	-2.26985	2.61907	2.31372
C	0.75581	2.26956	-3.20615	0.80046	2.01344	-3.37233	-1.02439	4.43282	3.33105	0.77735	4.51205	1.66693
O	0.09517	1.44218	-3.85916	0.07101	1.18206	-3.94339	-1.68679	5.11090	2.53415	1.79152	4.63951	2.34933
N	2.07551	2.15368	-3.01104	2.10839	1.81673	-3.17437	-1.36976	4.26909	4.62066	0.62331	5.10171	0.46004
N	1.81178	4.21702	-0.96555	2.04044	4.10234	-1.36263	0.56709	4.55956	0.60848	-2.07782	4.02909	0.32126
N	1.77740	4.70195	0.21385	2.06456	4.69583	-0.23349	1.41843	4.42882	-0.32938	-3.31084	3.77800	0.12953
N	2.88341	4.60213	0.87691	3.17700	4.58796	0.41718	1.15697	5.14058	-1.38552	-3.76499	4.11894	-1.03589
C	2.91058	5.12576	2.22897	3.26436	5.22820	1.71573	1.93799	4.86651	-2.57250	-5.20122	4.11171	-1.22097
C	4.05093	3.96037	0.30616	4.28798	3.81609	-0.10407	-0.06433	5.91388	-1.47425	-2.92132	4.71984	-2.04614
H	-2.50460	5.18194	-1.96488	-2.24000	5.22949	-2.34810	2.62907	1.71454	3.45345	-1.72304	1.55182	4.08189
H	-0.48576	5.73725	-0.50263	-0.14681	5.80155	-1.00123	2.58152	2.73700	1.10578	-3.17564	2.21834	2.10560
H	2.51572	2.75957	-2.32497	2.59951	2.44526	-2.54524	-0.80296	3.68414	5.21689	-0.24876	5.02970	-0.05027
H	2.48747	1.24319	-3.19070	2.45435	0.86740	-3.27679	-2.22441	4.67616	4.96614	1.35792	5.70568	0.12551
H	3.24673	4.33395	2.91223	3.56000	4.47991	2.46400	1.38633	4.18964	-3.24197	-5.59137	5.13970	-1.23986
H	3.59855	5.97957	2.29724	4.00842	6.03620	1.69704	2.16333	5.80557	-3.09122	-5.43514	3.60636	-2.16514
H	1.89928	5.45039	2.48914	2.28189	5.64276	1.95797	2.87330	4.38513	-2.26603	-5.65259	3.57385	-0.38115
H	4.80426	3.87015	1.09425	5.04932	3.75243	0.67910	-0.08149	6.41618	-2.44495	-3.43654	4.63258	-3.00735
H	3.75806	2.96200	-0.05081	3.92416	2.80931	-0.35749	-0.94285	5.25842	-1.37335	-2.71724	5.77716	-1.81817
H	4.43621	4.54737	-0.54001	4.69515	4.29091	-1.00822	-0.09847	6.65362	-0.66409	-1.96180	4.18898	-2.08408

Table S3. The geometrical parameters of hydrogen bonds X–H···A formed in the most stable complexes of TN:DTIC.

Comp.	Symb.	$d_{X\text{-}H}$ [Å]	$d_{H\cdots A}$ [Å]	$d_{X\ldots A}$ [Å]	∠X–H···A [°]
**TN:DTIC_1**	A	0.975	1.857	2.746	150.3
	B	0.974	1.950	2.883	159.5
	C	1.015	1.925	2.828	146.6
**TN:DTIC_2**	A	0.975	1.853	2.743	150.2
	B	0.975	1.883	2.833	164.0
	C	1.016	1.916	2.831	148.3
**TN:DTIC_3**	A	1.020	1.842	2.786	152.3
**TN:DTIC_4**	A	0.979	1.817	2.783	168.2

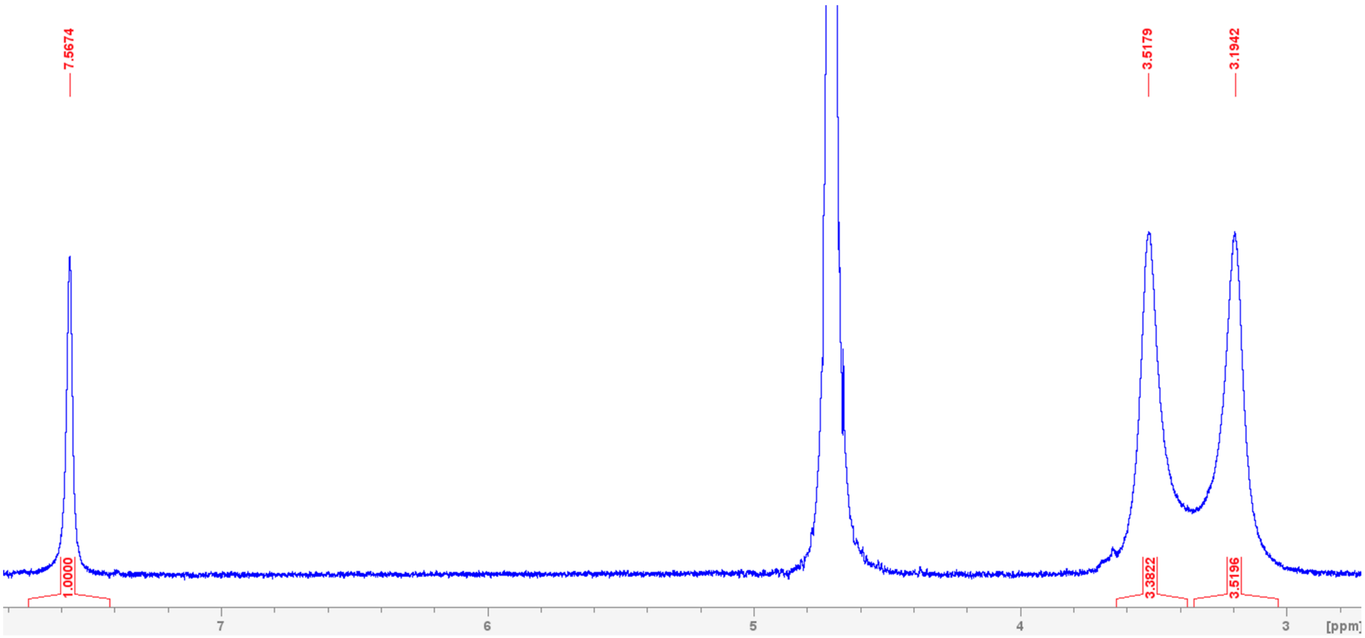

Figure S1. ^{1}H NMR spectrum of DTIC measured in D_2O.

^{1}H NMR (600MHz): 7.57(s, 1H, H-2); 3.52(sb, 3H, CH_3, NCH_3); 3.19(sb, 3H, CH_3, NCH_3)

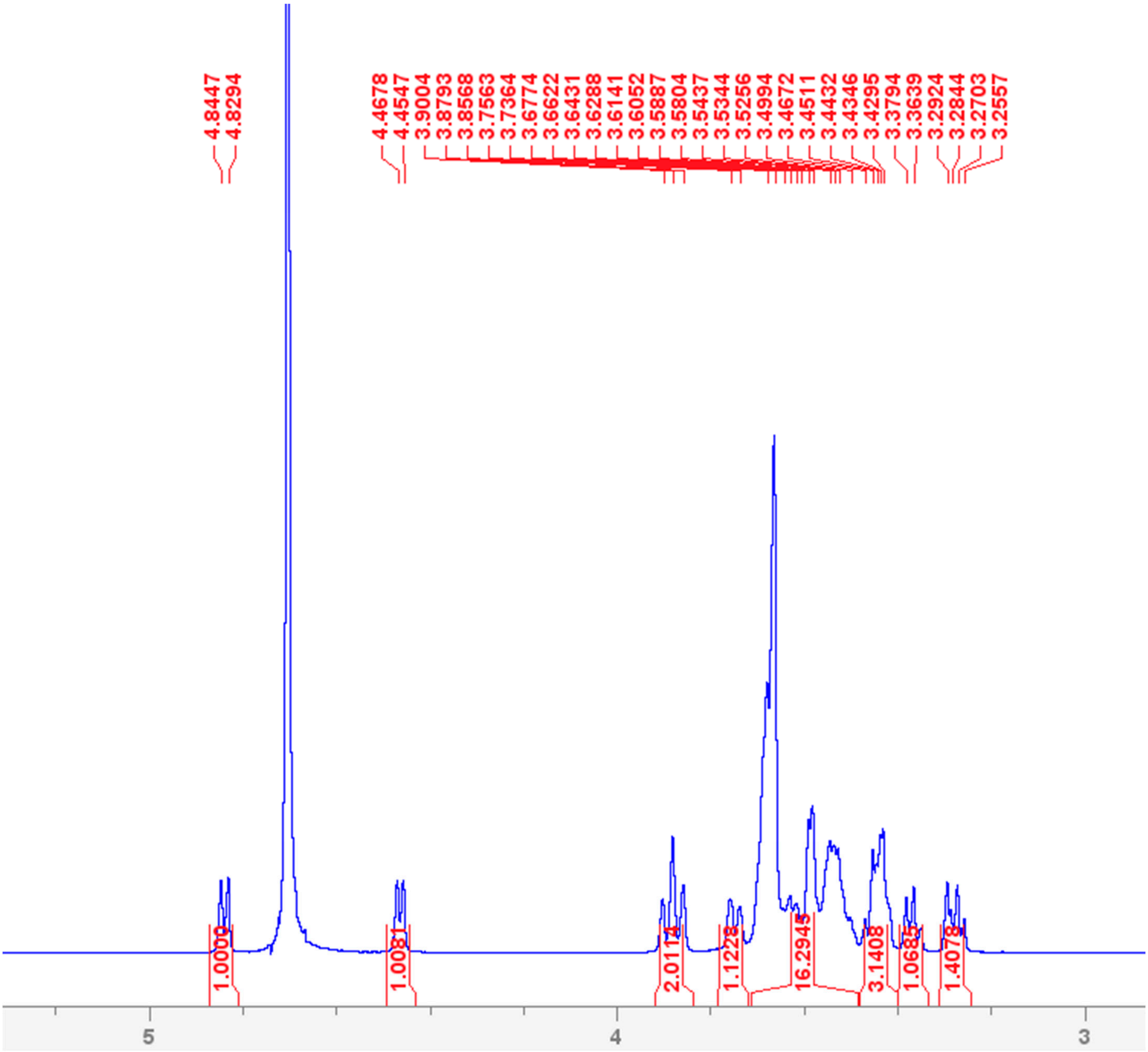

Figure S**2**. ^{1}H NMR spectrum of TN measured in D_2O.

^{1}H NMR (600MHz): 4.84(d, 2H, 2H-1, J=9.2 Hz); 4.46(d, 2H, 2H-1', J=7.9Hz); 3.92-3.84(m, 4H, 2H-6a, 2H-6'a); 3.78-3.72(m, 2H, 2H-5'); 3.71-3.60(m, 28H, 2H-6b, 2H-6'b, 12CH_2, crown); 3.69-3.57(m, 2H, 2H-4'); 3.56-3.48(m, 4H, 2H-4, 2H-2); 3.48-3.40(m, 4H, 2H-3', 2H-5); 3.39-3.34(m, 2H, 2H-3); 3.31-3.25(m, 2H, 2H-2').

Table S4. Chemical shifts of TN and DTIC protons in the free state and in the complex, along with the corresponding complexation-induced changes (Δδ). Measurements were performed in D_2O. Proton numbering is given in Figure 1 of the main article.

**TN protons**	**Chemical shift of TN**	**Chemical shift of TN in the complex**	**Δδ**	**DTIC protons**	**Chemical shift of DTIC**	**Chemical shift of DTIC in the complex**	**Δδ**
H-1	4.8371	4.8319	-0.0052	H-2	7.5674	7.5678	0.0004
H-1′	4.4613	4.4554	-0.0059	NCH_3	3.5179	-	-
H-6a	3.8793	3.8738	-0.0055	NCH_3	3.1942	3.2878	0.0936
H-6′a	3.8793	3.8738	-0.0055				
H-5′	3.7464	3.7405	-0.0059				
H-crown	3.6698	3.6614	-0.0084				
H-6b	3.6231	3.6162	-0.0069				
H-6′b	3.6231	3.6162	-0.0069				
H-4′	3.5804	3.5786	-0.0018				
H-4	3.5437	3.5357	-0.0080				
H-2	3.5256	3.5177	-0.0079				
H-3′	3.4511	3.4448	-0.0063				
H-5	3.4234	3.4183	-0.0051				
H-3	3.3639	3.3585	-0.0054				
H-2′	3.2703	3.2646	-0.0057				

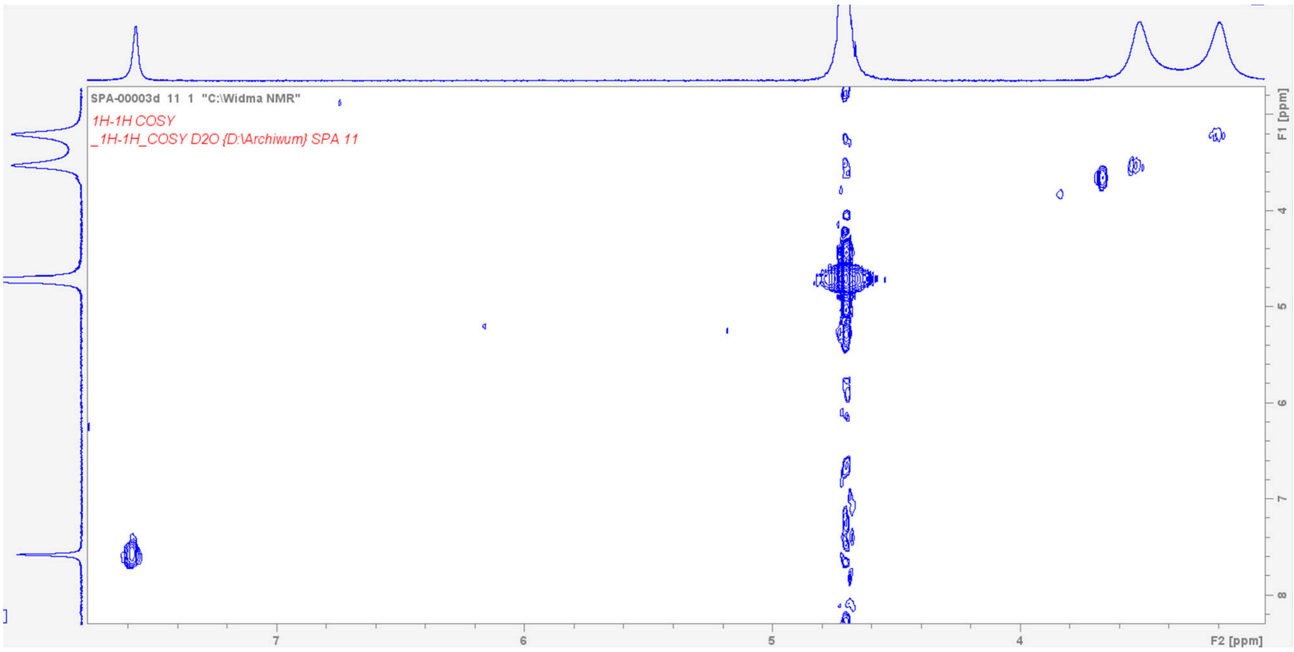

Figure S3. The COSY spectrum of DTIC.

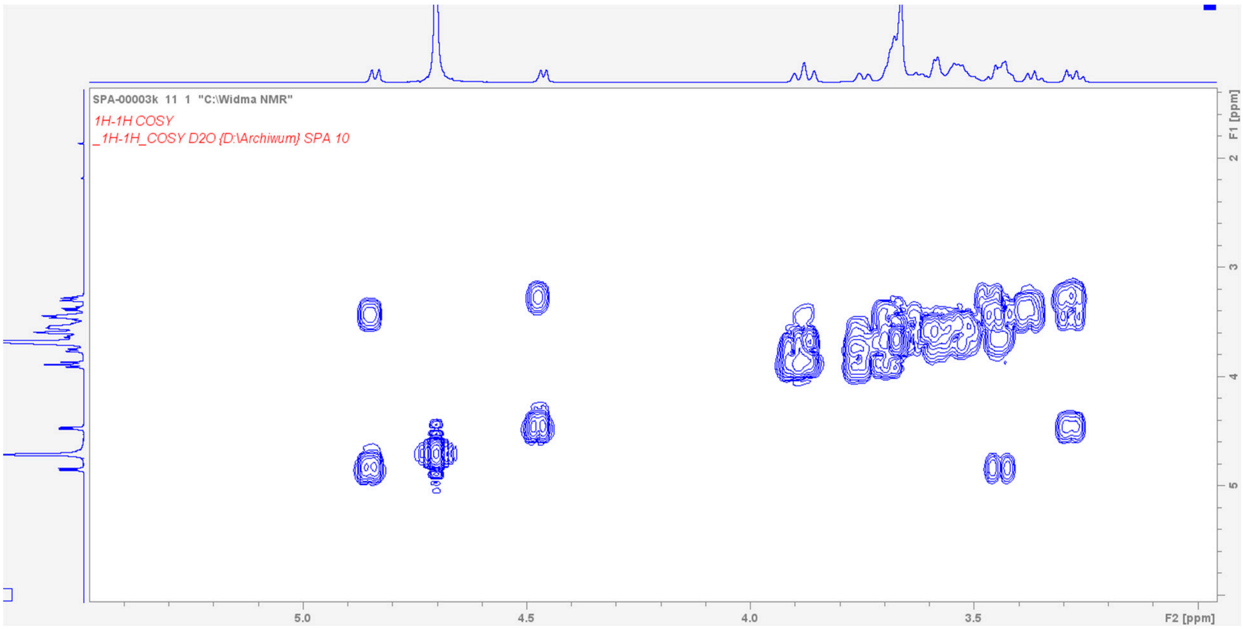

Figure S4. The COSY spectrum of TN.

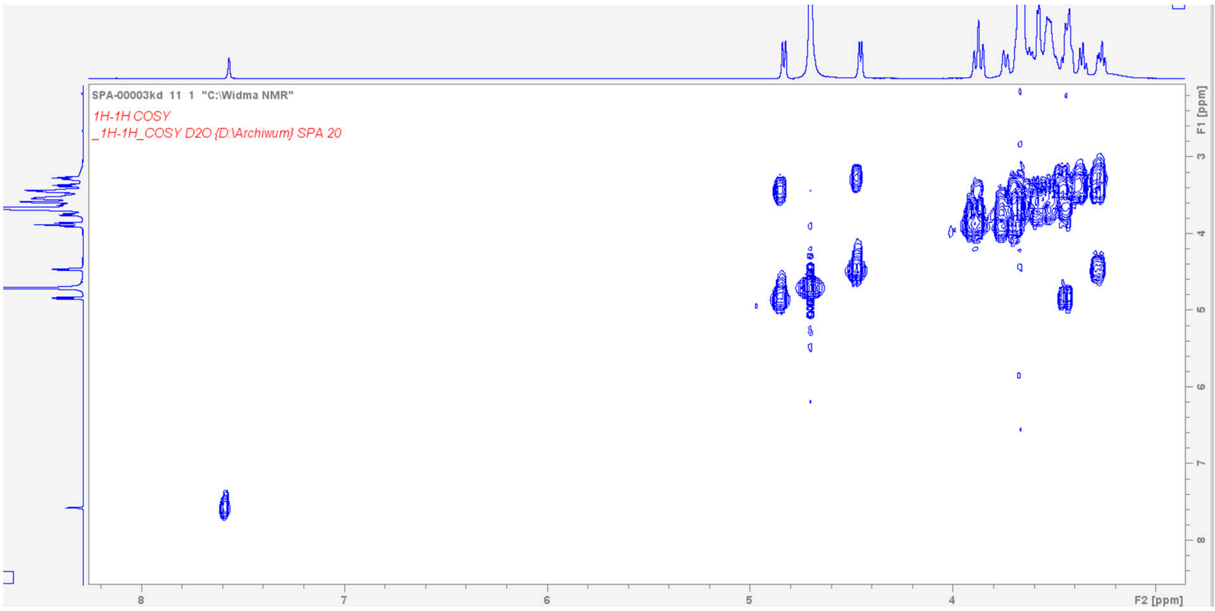

Figure S5. The COSY spectrum of the TN:DTIC complex.

Table S5. ^{1}H NMR chemical shifts values of TN in the TN:DTIC_1 complex obtained at the M08-HX/6-31++G(d,p) level of theory in water (PCM). CH_2 crown (C-N) and CH_2 crown (C-O) refer to protons in the diazacrown ether located near the N and O atoms, respectively. H-6a and H-6b denote the higher and lower chemical shift values of H-6, respectively. The same applies to H-6′a and H-6′b.

Protons (Figure 1 in the main article)	δ relative to TMS	δ scaled
CH_2 crown (C-N)	3.85	3.59
CH_2 crown (C-N)	3.00	2.86
CH_2 crown (C-N)	3.06	2.91
CH_2 crown (C-N)	3.81	3.56
CH_2 crown (C-N)	5.01	4.60
CH_2 crown (C-N)	2.69	2.59
CH_2 crown (C-N)	4.05	3.77
CH_2 crown (C-N)	2.90	2.77
CH_2 crown (C-O)	4.10	3.81
CH_2 crown (C-O)	3.43	3.23
CH_2 crown (C-O)	3.67	3.43
CH_2 crown (C-O)	3.80	3.55
CH_2 crown (C-O)	4.35	4.02
CH_2 crown (C-O)	5.14	4.71
CH_2 crown (C-O)	4.13	3.84
CH_2 crown (C-O)	4.12	3.83
CH_2 crown (C-O)	3.44	3.24
CH_2 crown (C-O)	4.10	3.81
CH_2 crown (C-O)	4.91	4.51
CH_2 crown (C-O)	3.03	2.89
CH_2 crown (C-O)	4.27	3.95
CH_2 crown (C-O)	3.33	3.15
CH_2 crown (C-O)	4.16	3.86
CH_2 crown (C-O)	3.98	3.71
H-1	4.81	4.43
H-1	6.15	5.58
H-2	3.78	3.54
H-2	2.96	2.82
H-3	2.66	2.57
H-3	4.51	4.17
H-4	4.36	4.03
H-4	3.21	3.04
H-5	3.21	3.04
H-5	4.51	4.16
H-6a	4.44	4.11
H-6b	3.80	3.55
H-6b	3.61	3.38

H-6a	4.59	4.23
H-1'	5.64	5.14
H-1'	4.26	3.95
H-2'	3.16	3.00
H-2'	4.27	3.96
H-3'	4.07	3.79
H-3'	3.41	3.21
H-4'	3.01	2.86
H-4'	3.45	3.25
H-5'	4.24	3.93
H-5'	3.66	3.43
H-6'b	3.76	3.52
H-6'a	3.97	3.70
H-6'a	5.02	4.60
H-6'b	3.47	3.27

Table S6. ^{1}H NMR chemical shifts values of TN in the TN:DTIC_2 complex obtained at the M08-HX/6-31++G(d,p) level of theory in water (PCM).

Protons (Figure 1 in the main article)	δ relative to TMS	δ scaled
CH_2 crown (C-N)	3.96	3.69
CH_2 crown (C-N)	2.97	2.83
CH_2 crown (C-N)	3.05	2.90
CH_2 crown (C-N)	3.72	3.48
CH_2 crown (C-N)	4.84	4.45
CH_2 crown (C-N)	2.67	2.57
CH_2 crown (C-N)	4.00	3.72
CH_2 crown (C-N)	2.99	2.85
CH_2 crown (C-O)	4.05	3.77
CH_2 crown (C-O)	3.48	3.27
CH_2 crown (C-O)	3.89	3.63
CH_2 crown (C-O)	3.48	3.27
CH_2 crown (C-O)	4.18	3.88
CH_2 crown (C-O)	5.11	4.68
CH_2 crown (C-O)	4.11	3.82
CH_2 crown (C-O)	4.18	3.88
CH_2 crown (C-O)	3.41	3.22
CH_2 crown (C-O)	4.09	3.80
CH_2 crown (C-O)	4.92	4.52
CH_2 crown (C-O)	3.13	2.97
CH_2 crown (C-O)	4.22	3.91
CH_2 crown (C-O)	3.36	3.17
CH_2 crown (C-O)	4.10	3.81

CH_2 crown (C-O)	4.02	3.74
H-1	4.95	4.54
H-1	5.89	5.35
H-2	3.80	3.55
H-2	3.08	2.93
H-3	2.49	2.42
H-3	4.23	3.92
H-4	3.89	3.63
H-4	3.31	3.12
H-5	3.11	2.96
H-5	4.67	4.30
H-6a	4.37	4.04
H-6b	3.63	3.41
H-6b	3.38	3.18
H-6a	4.38	4.05
H-1'	5.38	4.92
H-1'	4.71	4.34
H-2'	3.19	3.02
H-2'	4.21	3.90
H-3'	4.05	3.76
H-3'	3.39	3.19
H-4'	2.85	2.73
H-4'	3.59	3.37
H-5'	3.79	3.54
H-5'	3.91	3.65
H-6'b	3.94	3.67
H-6'a	3.86	3.60
H-6'a	4.92	4.52
H-6'b	3.51	3.30

Table S7. ^{1}H NMR chemical shifts values of TN in the TN:DTIC_3 complex obtained at the M08-HX/6-31++G(d,p) level of theory in water (PCM).

Protons (Figure 1 in the main article)	δ relative to TMS	δ scaled
CH_2 crown (C-N)	3.92	3.66
CH_2 crown (C-N)	2.77	2.66
CH_2 crown (C-N)	3.03	2.88
CH_2 crown (C-N)	3.71	3.48
CH_2 crown (C-N)	5.04	4.62
CH_2 crown (C-N)	2.64	2.55
CH_2 crown (C-N)	3.96	3.69
CH_2 crown (C-N)	2.93	2.80
CH_2 crown (C-O)	4.17	3.87
CH_2 crown (C-O)	3.51	3.30

CH_2 crown (C-O)	3.71	3.47
CH_2 crown (C-O)	3.64	3.41
CH_2 crown (C-O)	4.68	4.31
CH_2 crown (C-O)	5.19	4.75
CH_2 crown (C-O)	4.04	3.76
CH_2 crown (C-O)	4.05	3.76
CH_2 crown (C-O)	3.33	3.15
CH_2 crown (C-O)	3.99	3.71
CH_2 crown (C-O)	4.95	4.54
CH_2 crown (C-O)	3.21	3.04
CH_2 crown (C-O)	4.36	4.03
CH_2 crown (C-O)	3.32	3.14
CH_2 crown (C-O)	4.22	3.91
CH_2 crown (C-O)	4.01	3.74
H-1	5.03	4.62
H-1	6.28	5.69
H-2	3.76	3.52
H-2	3.04	2.90
H-3	3.31	3.12
H-3	4.19	3.89
H-4	4.27	3.95
H-4	3.02	2.88
H-5	3.47	3.27
H-5	4.23	3.92
H-6a	4.34	4.02
H-6b	3.79	3.54
H-6b	3.70	3.47
H-6a	4.33	4.01
H-1'	5.58	5.09
H-1'	3.98	3.71
H-2'	3.37	3.18
H-2'	4.23	3.92
H-3'	4.15	3.85
H-3'	3.48	3.27
H-4'	2.75	2.64
H-4'	3.57	3.35
H-5'	4.24	3.93
H-5'	3.86	3.60
H-6'a	3.93	3.66
H-6'a	4.07	3.78
H-6'b	3.83	3.58
H-6'b	3.51	3.30

Table S8. ^{1}H NMR chemical shifts values of TN in the TN:DTIC_4 complex obtained at the M08-HX/6-31++G(d,p) level of theory in water (PCM).

Proton (Figure 1 in the main article)	δ relative to TMS	δ scaled
CH_2 crown (C-N)	3.78	3.53
CH_2 crown (C-N)	3.07	2.92
CH_2 crown (C-N)	3.02	2.88
CH_2 crown (C-N)	3.88	3.62
CH_2 crown (C-N)	5.18	4.74
CH_2 crown (C-N)	2.66	2.57
CH_2 crown (C-N)	3.97	3.70
CH_2 crown (C-N)	2.94	2.81
CH_2 crown (C-O)	4.10	3.81
CH_2 crown (C-O)	3.46	3.26
CH_2 crown (C-O)	3.63	3.40
CH_2 crown (C-O)	3.74	3.50
CH_2 crown (C-O)	4.28	3.97
CH_2 crown (C-O)	4.88	4.48
CH_2 crown (C-O)	3.93	3.66
CH_2 crown (C-O)	4.04	3.76
CH_2 crown (C-O)	3.46	3.26
CH_2 crown (C-O)	4.13	3.84
CH_2 crown (C-O)	4.68	4.31
CH_2 crown (C-O)	3.25	3.07
CH_2 crown (C-O)	4.35	4.03
CH_2 crown (C-O)	3.30	3.11
CH_2 crown (C-O)	4.25	3.94
CH_2 crown (C-O)	3.91	3.64
H-1	4.87	4.48
H-1	6.74	6.09
H-2	3.67	3.44
H-2	2.81	2.70
H-3	3.48	3.27
H-3	4.02	3.74
H-4	4.62	4.26
H-4	3.04	2.89
H-5	3.50	3.29
H-5	4.63	4.27
H-6a	4.19	3.89
H-6a	3.70	3.47
H-6b	3.72	3.48
H-6b	4.15	3.86
H-1'	5.33	4.87

H-1'	4.13	3.83
H-2'	3.49	3.28
H-2'	4.31	3.99
H-3'	3.82	3.57
H-3'	3.34	3.15
H-4'	3.05	2.91
H-4'	3.63	3.40
H-5'	3.87	3.61
H-5'	3.88	3.62
H-6'a	3.81	3.56
H-6'a	3.96	3.69
H-6'b	4.77	4.39
H-6'b	3.49	3.28

Table S9. The value of molar concentration of salt C_{DTIC} [mol/dm³], concentration of ligand C_{TN} [mol/dm³], molar conductivity Λ_m [S·cm²·mol⁻¹] for TN with DTIC in water at all tested temperatures at pressure p = 0.1 MPa.[a]

T [K]			293.15	298.15	303.15	308.15	313.15
Nr	C_{DTIC} mol/dm³]	C_{TN} [mol/dm³]	Λ_m [S • cm² • mol⁻¹]	Λ_m [S • cm² • mol⁻¹]	Λ_m [S • cm² • mol⁻¹]	Λ_m [S • cm² • mol⁻¹]	Λ_m [S • cm² • mol⁻¹]
1.	0.002871	0.000000	40.1292	44.3792	47.1826	50.2043	53.0278
2.	0.002815	0.000298	39.1378	43.3877	46.1912	49.2129	52.0364
3.	0.002761	0.000577	38.1365	42.3864	45.1899	48.2116	51.0351
4.	0.002704	0.000876	37.0824	41.3324	44.1359	47.1575	49.9811
5.	0.002640	0.001212	35.9511	40.2010	43.0045	46.0262	48.8497
6.	0.002589	0.001479	35.0828	39.3327	42.1362	45.1579	47.9814
7.	0.002533	0.001777	34.1538	38.4038	41.2073	44.2289	47.0525
8.	0.002476	0.002074	33.5125	37.7625	40.5660	43.5876	46.4112
9.	0.002419	0.002373	32.8295	37.0795	39.8829	42.9046	45.7282
10.	0.002364	0.002663	32.1853	36.4353	39.2388	42.2604	45.0840
11.	0.002310	0.002944	31.6579	35.9079	38.7113	41.7330	44.5565
12.	0.002253	0.003244	31.1226	35.3726	38.1760	41.1977	44.0212
13.	0.002196	0.003545	30.6355	34.8855	37.6890	40.7106	43.5342
14.	0.002137	0.003851	30.2317	34.4816	37.2851	40.3067	43.1303
15.	0.002079	0.004156	29.8894	34.1394	36.9429	39.9645	42.7881
16.	0.002021	0.004458	29.6504	33.9003	36.7038	39.7255	42.5490
17.	0.001967	0.004746	29.3985	33.6485	36.4519	39.4736	42.2971
18.	0.001911	0.005039	29.2280	33.4779	36.2814	39.3030	42.1266
19.	0.001853	0.005342	28.9994	33.2493	36.0528	39.0745	41.8980
20.	0.001798	0.005628	28.8280	33.0779	35.8814	38.9031	41.7266
21.	0.001743	0.005920	28.7139	32.9639	35.7673	38.7890	41.6125
22.	0.001683	0.006235	28.5547	32.8047	35.6081	38.6298	41.4533
23.	0.001631	0.006508	28.4441	32.6941	35.4975	38.5192	41.3427

[a] Standard uncertainties are $u(T)$ = 0.01 K, $u(p)$ = 0.05 MPa, $u(c) = 10^{-4} \cdot c$, and the combined expanded uncertainty is $U_c(\Lambda) = 0.0005 \cdot \Lambda$ (level of confidence = 0.95).

Table S10. Theoretical values of the Gibbs free energy (ΔG) [kJ/mol] obtained for the most stable complexes at the M08-HX-D3/6-31G(d,p) level of theory in water (PCM).

**Temp.**	**TN:DTIC_1**	**TN:DTIC_2**	**TN:DTIC_3**	**TN:DTIC_4**
293	-30.89	-26.52	-36.92	-19.63
298	-29.96	-25.58	-36.01	-18.63
303	-29.01	-24.64	-35.12	-17.68
308	-28.08	-23.70	-34.22	-16.61
313	-27.15	-22.77	-33.33	-15.61

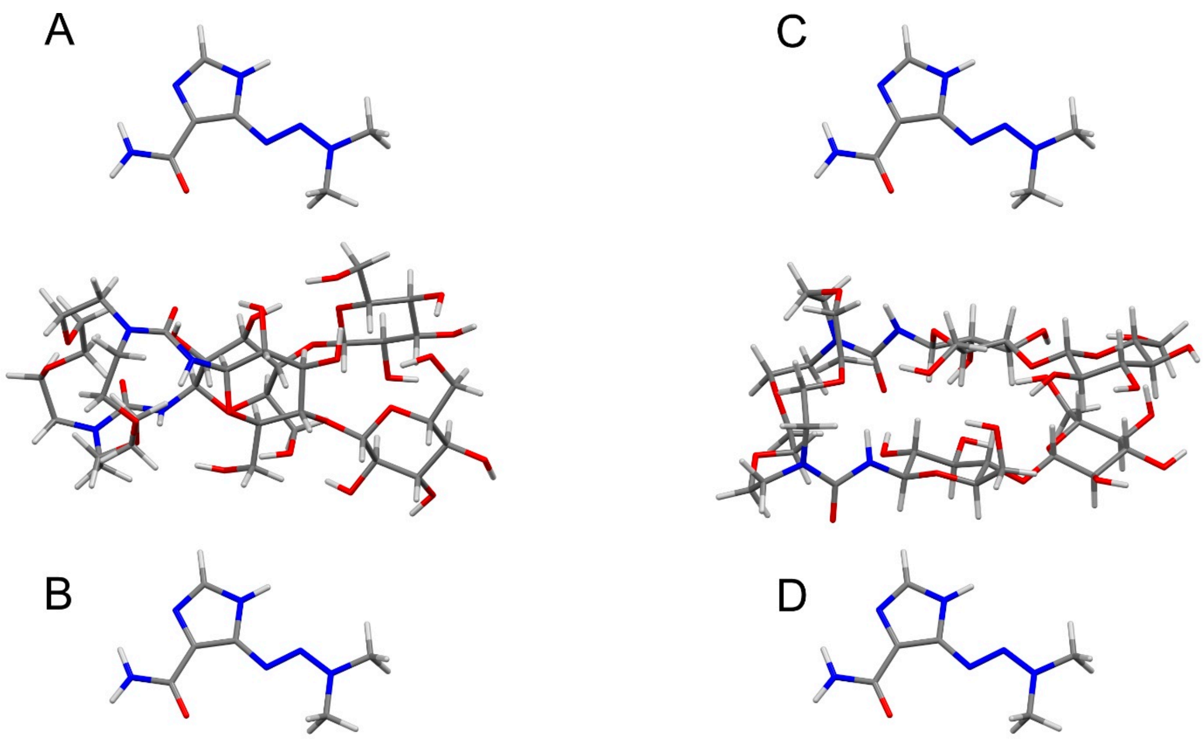

Figure S6. Initial model of the complexes considered during the configurational search. In configurations C and D, TN is rotated around the X axis by approximately 90° relative to its orientation in configuration A.

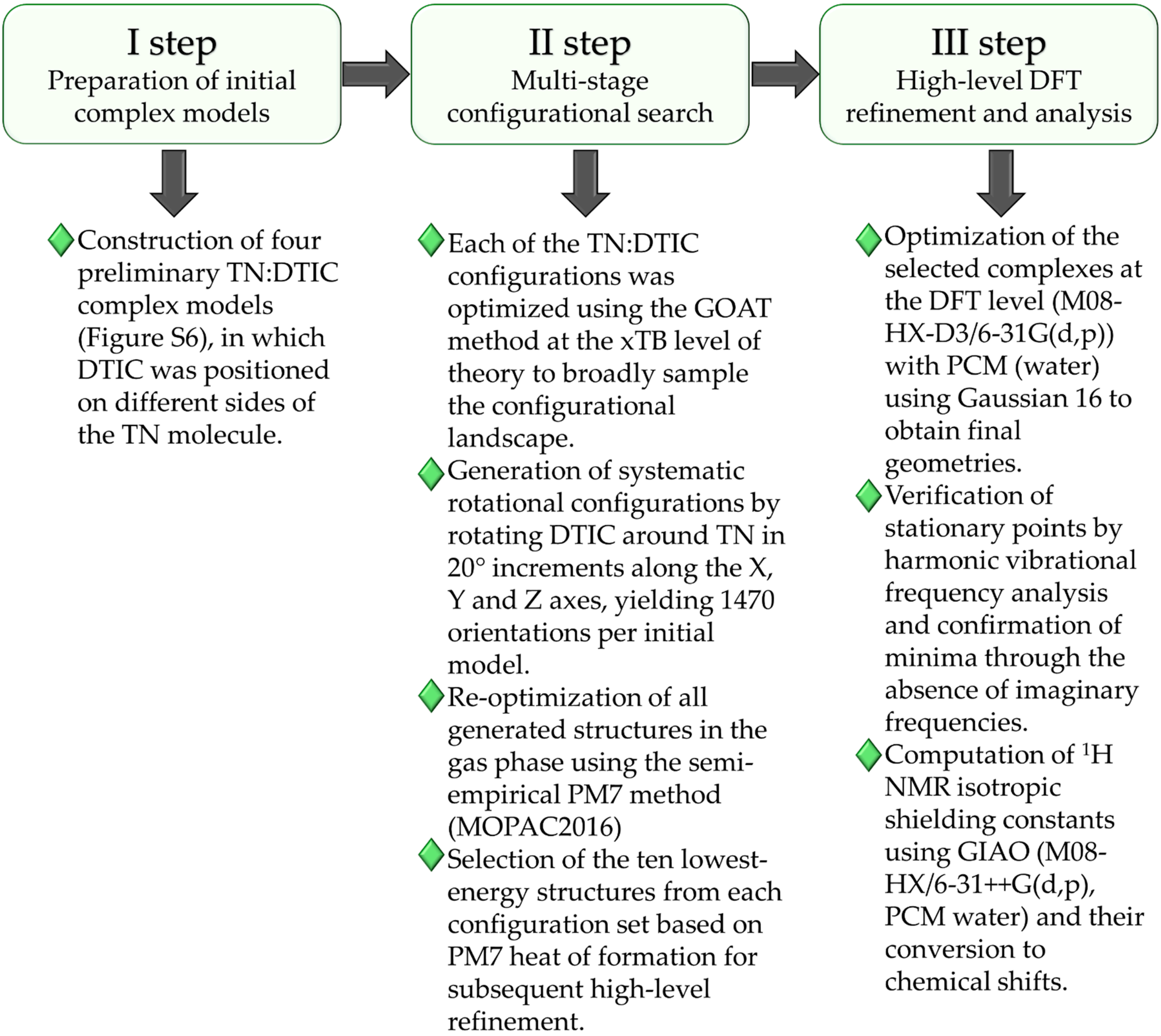

Chart S1. Flowchart illustrating the computational analysis carried out in this study.
